# Polyphenol-Rich *Opuntia ficus-indica* Cladodes: An Integrated Metabolomic, In Vivo and In Silico Study Supporting Their Hypolipidemic and Hepatoprotective Effects

**DOI:** 10.3390/nu18172766

**Published:** 2026-08-24

**Authors:** Abderrahmane Hadini, Abdelhay Addous, Abdellah Baraich, Mourad Bendada, Ahmed Karim, Mohammed Choukri, Imane Mokhtari, Rémy Cordazzo, Pierre Pétriacq, Souliman Amrani, Anthony Bernard, Khalid El Bekkaye, Luca Rastrelli, Maria D’Elia, Hicham Harnafi

**Affiliations:** 1 Laboratory of Bioresources, Biotechnologies, and Sustainable Development, Faculty of Sciences, Mohammed First University, Oujda 60000, Morocco; abderrahmane.hadini@ump.ac.ma (A.H.); abdellah.baraich@ump.ac.ma (A.B.); mokhtari.imane@ump.ac.ma (I.M.); s.amrani@ump.ac.ma (S.A.); 2 Laboratory of Pharmacology, Genomics, Environment and Health, Faculty of Sciences, Mohammed First University, Oujda 60000, Morocco; abdelhay.addous.d23@ump.ac.ma (A.A.); a.karim@ump.ac.ma (A.K.); 3 Laboratory of the Improvement of Agricultural Production, Biotechnology and the Environment, Faculty of Sciences, Mohammed First University, Oujda 60000, Morocco; mouradbendada4@gmail.com (M.B.); elbekkaye2015@gmail.com (K.E.B.); 4 Biochemistry Laboratory, Central Laboratory Service, University Hospital Center, Oujda 60000, Morocco; m.choukri@ump.ac.ma; 5 UMR1332 BFP, University of Bordeaux, INRAE, 33140 Villenave d’Ornon, France; remy.cordazzo@inrae.fr (R.C.); pierre.petriacq@inrae.fr (P.P.); anthony.bernard@inrae.fr (A.B.); 6 Bordeaux Metabolome, MetaboHUB, PHENOME-EMPHASIS, 33140 Villenave d’Ornon, France; 7 Dipartimento di Farmacia, University of Salerno, Via Giovanni Paolo II, 132, Fisciano, 84084 Salerno, Italy; mdelia@unisa.it; 8 National Biodiversity Future Center (NBFC), 90133 Palermo, Italy; 9 Dipartimento di Scienze della Terra e del Mare, University of Palermo, 90123 Palermo, Italy

**Keywords:** *Opuntia ficus-indica*, cladodes, hyperlipidemia, dietary bioactive compounds, untargeted metabolomics, lipid metabolism, molecular docking, oxidative stress

## Abstract

**Background:** Hyperlipidemia is a major risk factor for cardiometabolic disorders, including non-alcoholic fatty liver disease (NAFLD), and is closely associated with oxidative stress. *Opuntia ficus-indica* (OFI) cladodes are recognized as a rich source of bioactive phytochemicals; however, the molecular mechanisms underlying their metabolic benefits remain incompletely understood. **Objectives:** This study aimed to comprehensively evaluate the hypolipidemic and hepatoprotective potential of a polyphenol-rich *O. ficus-indica* cladode extract (OCE) using an integrated approach combining in vivo evaluation, untargeted metabolomics (UHPLC-Orbitrap-MS/MS), molecular docking, and ADMET prediction. **Methods:** Hyperlipidemic mice fed a high-fat diet (HFD) were treated with OCE, while molecular docking was performed on ten major annotated phytochemicals against twelve key proteins involved in lipid metabolism and cholesterol homeostasis, including HMGCR, FAS, PPARα, PCSK9, and NPC1L1, using simvastatin as the reference compound. **Results:** OCE treatment significantly improved plasma and hepatic lipid profiles, improved glucose homeostasis, and markedly reduced hepatic malondialdehyde (MDA) levels, indicating attenuation of oxidative stress. Histopathological analysis further supported a pronounced hepatoprotective effect, with a substantial reduction in hepatic steatosis. Untargeted metabolomics enabled the annotation of 102 metabolites, putatively identifying piscidic acid as the predominant phenolic constituent together with a diverse profile of flavonoids and phenolic acids. Molecular docking supported the potential contribution of these phytochemicals to the regulation of lipid metabolism through favorable interactions with multiple therapeutic targets, while ADMET prediction suggested an overall favorable pharmacokinetic and toxicity profile despite the lower intestinal permeability predicted for glycosylated derivatives. **Conclusions:** Overall, these findings support *O. ficus-indica* cladodes as a promising source of dietary bioactive compounds with potential applications in the nutritional management and prevention of hyperlipidemia and related cardiometabolic disorders.

## 1. Introduction

Cholesterol, triglycerides (TG), and lipoproteins (LPs) are essential lipid components that play fundamental roles in maintaining cellular structure and function. Cholesterol contributes to membrane integrity and fluidity, participates in lipid raft formation, and serves as the precursor of steroid hormones, bile acids, vitamin D, and oxysterols, thereby regulating numerous physiological processes [1,2]. Cellular cholesterol homeostasis is tightly controlled through a dynamic balance between hepatic de novo synthesis, dietary uptake, intracellular storage, and transport [3]. Disruption of this balance leads to hyperlipidemia (HLP), a metabolic disorder characterized by elevated plasma concentrations of total cholesterol (TC), TG, and low-density lipoprotein cholesterol (LDL-C), together with reduced high-density lipoprotein cholesterol (HDL-C). Persistent dyslipidemia promotes atherosclerosis and markedly increases the risk of cardiovascular disease (CVD), which represents one of the leading causes of global morbidity and mortality [4,5].

Cardiovascular diseases account for approximately 17.9 million deaths annually worldwide, corresponding to nearly one-third of all deaths, and are largely associated with unhealthy dietary habits, physical inactivity, obesity, smoking, and other lifestyle-related risk factors [6]. In Morocco, CVDs are responsible for approximately 38% of all deaths, emphasizing their major public health burden [7]. Beyond cardiovascular complications, hyperlipidemia is closely associated with metabolic dysfunction-associated steatotic liver disease (MASLD), formerly referred to as non-alcoholic fatty liver disease, which may progressively evolve into fibrosis, cirrhosis, and hepatocellular carcinoma [8,9]. Dyslipidemia is also associated with type 2 diabetes mellitus, chronic kidney disease, and several extrahepatic malignancies [10,11]. Moreover, excessive lipid accumulation promotes oxidative stress through increased production of reactive oxygen species (ROS), lipid peroxidation, and oxidation of LDL particles (ox-LDL), thereby activating inflammatory pathways, including NF-κB signaling and the production of pro-inflammatory cytokines such as interleukin-6 (IL-6) [12,13].

Current pharmacological management of hyperlipidemia mainly relies on lipid-lowering agents aimed at reducing cardiovascular risk [14]. Statins, particularly simvastatin, remain the first-line therapy because they inhibit 3-hydroxy-3-methylglutaryl-coenzyme A (HMG-CoA) reductase, the rate-limiting enzyme in cholesterol biosynthesis [15]. Other therapeutic options include fibrates, which activate peroxisome proliferator-activated receptor alpha (PPARα) to reduce circulating triglycerides, as well as newer approaches targeting cholesterol metabolism, such as PCSK9 inhibitors and small interfering RNA (siRNA)-based therapies (e.g., inclisiran), which enhance LDL receptor availability and lower circulating LDL-C concentrations [16]. In addition, several proteins involved in cholesterol absorption, transport, and efflux, including NPC1L1, ABCA1, CETP, and LDLR, have emerged as attractive therapeutic targets [17].

Although these pharmacological strategies are highly effective, long-term treatment may be associated with adverse effects, including myopathy, hepatotoxicity, nephrotoxicity, and, more rarely, severe muscle injury [18]. Consequently, increasing attention has been devoted to identifying dietary strategies and plant-derived bioactive compounds capable of complementing conventional therapies. Medicinal and edible plants represent an important source of phytochemicals with antioxidant, anti-inflammatory, and lipid-lowering activities, making them promising candidates for the nutritional management of cardiometabolic disorders [17,19,20]. In this context, the Mediterranean region, particularly Morocco, represents an important reservoir of plant biodiversity characterized by numerous species with recognized nutritional and therapeutic value [21,22].

Among these, the cactus pear (*Opuntia* spp.; Cactaceae) has attracted increasing scientific interest owing to its nutritional composition and wide range of biological activities. Native to Mexico and currently cultivated worldwide, the genus comprises more than 300 species that are extensively used in the food, nutraceutical, pharmaceutical, and cosmetic industries while also contributing to ecosystem sustainability [23]. In Morocco, especially in the eastern region, *Opuntia ficus-indica* (L.) Mill. has been developed as a strategic agricultural crop due to its remarkable adaptation to arid and semi-arid environments. Its Crassulacean Acid Metabolism (CAM) enables it to achieve high water-use efficiency and remarkable drought tolerance, which is the reason for its widespread promotion in national agricultural and reforestation programs for desertification control and climate resilience enhancement [24,25,26]. In addition to its agronomic value, *O. ficus-indica* has long been known for its nutritional and medicinal value. The edible cladodes are traditionally consumed fresh, or in the form of decoctions and juices, for the management of various metabolic ailments [27,28,29]. Pharmacologically, *O. ficus-indica* cladode extracts have been shown in many in vitro and in vivo studies to exhibit potent antioxidant and radical-scavenging properties, reduce hepatic oxidative stress, improve glycemic control, and exert marked anti-inflammatory and hepatoprotective effects [30,31,32,33]. In particular, previous studies have specifically demonstrated that cladode extracts can modulate cholesterol metabolism, including in HepG2 cell models, providing additional support for their potential in the management of hyperlipidemia [34]. More recently, our metabolomic investigation revealed that *Opuntia* cladodes are particularly rich in phenolic acids and flavonoids, including piscidic acid, xanthorhamnin, and isorhamnetin-3-*O*-rutinoside, together with carboxylic acids, amino acids, fatty acids, sugars, and dietary fibers. Moreover, these extracts exhibited remarkable antioxidant properties without showing genotoxic effects in experimental models [35].

Building upon these findings, the present study aimed to evaluate the hypolipidemic, antioxidant and hepatoprotective effects of aqueous extracts obtained from *O. ficus-indica* cladodes collected in eastern Morocco using a high-fat-diet (HFD)-induced hyperlipidemic mouse model. Plasma lipid parameters (TC, TG, HDL-C, and LDL-C), glycemia, hepatic oxidative stress assessed by malondialdehyde (MDA) determination, acute toxicity, and liver histopathology were evaluated, with simvastatin used as the reference drug. To comprehensively characterize the extract used in the biological experiments, untargeted UHPLC–Orbitrap MS/MS metabolomics was combined with in vivo pharmacological evaluation to investigate its chemical composition and biological effects. Molecular docking and computational ADMET analysis were carried out to explore potential molecular interactions and provide mechanistic insights. Overall, this integrated approach was intended to link the phytochemical composition of OCE to its observed metabolic effects, and to identify candidate metabolites and molecular targets for subsequent experimental validation.

## 2. Materials and Methods

### 2.1. Chemicals and Reagents

Analytical-grade reagents were used throughout the study. Folin–Ciocalteu reagent, gallic acid, rutin, catechin, sodium carbonate, sodium nitrite, aluminum chloride, sodium hydroxide, vanillin, hydrochloric acid, DPPH, ascorbic acid, methanol, ethanol, sulfuric acid, sodium phosphate, ammonium molybdate, trichloroacetic acid, thiobarbituric acid, trisodium citrate, *n*-hexane, ethyl acetate, isopropanol, acetonitrile, formic acid, hematoxylin, eosin, toluene, paraffin wax, and simvastatin were purchased from Sigma-Aldrich (St. Louis, MO, USA), unless otherwise specified. Commercial enzymatic kits for total cholesterol, triglycerides, HDL-C, LDL-C, and glucose were obtained from BioSystems S.A. (Barcelona, Spain). Ultrapure or deionized distilled water was used for all preparations, and solutions were freshly prepared before each experiment.

### 2.2. Preparation of O. ficus-indica Cladode Extract (OCE)

Cladodes of *Opuntia ficus-indica* (L.) Mill. were collected in April 2025 from Guenfouda (34°27′29.42″ N, 2°3′43.28″ W), Jerada Province, Eastern Region, Morocco. Plant material was authenticated by Dr. Abdelmonaim Homrani Bakali, researcher and botanist at the National Institute of Agronomic Research (INRA), Errachidia, Morocco. A voucher specimen was deposited in the Herbarium of Mohammed First University, Oujda, Morocco (HUMPOM; Voucher No. 37).

Fresh cladodes were washed with distilled water, manually cleaned to remove spines, shade-dried at approximately 25 °C for one week, cut into small pieces, and further dried in a ventilated oven at 40 °C for 48 h. The dried material was ground using an electric grinder (Model 0405 Silver, Moulinex, Écully, France) and sieved through a 120-mesh sieve (125 µm). The resulting powder was stored in airtight containers protected from light until extraction.

The powdered cladodes were extracted with hot distilled water at a solid-to-solvent ratio of 1:10 (*w*/*v*), followed by sonication for 40 min. The mixture was filtered through Whatman filter paper, and the resulting aqueous extract was frozen and lyophilized using a freeze dryer (Alpha 1–2 LD, Christ, Germany) until complete dryness. The dried crude extract (OCE) was stored at −20 °C, protected from light, until further analyses.

### 2.3. Liquid–Liquid Fractionation of OCE

Liquid–liquid fractionation was performed by dissolving 2 g of OCE in 50 mL of distilled water in a separatory funnel. The aqueous solution was first defatted by three successive extractions with 50 mL of *n*-hexane to remove lipophilic compounds, including chlorophylls and lipids. The remaining aqueous phase was subsequently extracted three times with 50 mL of ethyl acetate.

The ethyl acetate fraction was concentrated to dryness under reduced pressure using a rotary evaporator (Büchi Rotavapor R-300, Büchi Labortechnik AG, Flawil, Switzerland), whereas the residual aqueous fraction was frozen and lyophilized using a freeze dryer (Alpha 1–2 LD, Christ, Germany) until complete dryness. The resulting dried fractions were stored at −20 °C, protected from light, until further analysis.

The extraction yield was calculated as follows (Equation (1)):

(1) $$\mathrm{Yield}\left(\%\right)=\frac{\mathrm{mass}\;\mathrm{of}\;\mathrm{the}\;\mathrm{dry}\;\mathrm{fracti}}{\mathrm{mass}\;\mathrm{of}\;\mathrm{the}\;\mathrm{cladodes}\;\mathrm{dry}\;\mathrm{extract}}\;*\;100$$

### 2.4. Determination of Total Phenolic, Flavonoid, and Condensed Tannin Contents

The total phenolic content (TPC) was determined using the Folin–Ciocalteu colorimetric assay with slight modifications, as previously described by Hadini et al. [36]. Briefly, 250 μL of OCE was mixed with 1.87 mL of distilled water and 125 μL of Folin–Ciocalteu reagent. After 5 min, 250 μL of 20% (*w*/*v*) sodium carbonate solution was added, and the mixture was incubated in the dark at room temperature for 30 min. Absorbance was measured at 760 nm using a microplate reader (BioTek Gen5, BioTek Instruments Inc., Winooski, VT, USA). TPC was quantified using a gallic acid calibration curve and expressed as milligrams of gallic acid equivalents per gram of dry matter (mg GAE/g DM).

Total flavonoid content (TFC) was determined using the aluminum chloride colorimetric method with slight modifications [37]. Briefly, 500 μL of OCE was mixed with 3 mL of distilled water and 300 μL of 5% (*w*/*v*) sodium nitrite. After 5 min of incubation in the dark, 300 μL of 10% (*w*/*v*) aluminum chloride solution was added and allowed to react for an additional 5 min. Subsequently, 2 mL of 1 M sodium hydroxide was added, and the final volume was adjusted to 10 mL with distilled water. After incubation for 15 min in the dark, absorbance was measured at 430 nm. TFC was calculated using a rutin calibration curve and expressed as milligrams of rutin equivalents per gram of dry matter (mg RE/g DM).

Condensed tannin content was determined using the vanillin assay, as previously described by Bendada et al. [38]. Briefly, 50 μL of OCE was mixed with 1.5 mL of 4% (*w*/*v*) vanillin solution prepared in ethanol and 750 μL of concentrated hydrochloric acid. After incubation at 25 °C for 20 min, absorbance was measured at 550 nm. Condensed tannin content was calculated using catechin as the reference standard and expressed as milligrams of catechin equivalents per gram of dry matter (mg CE/g DM).

### 2.5. UHPLC–Orbitrap IQ-X Tribrid MS/MS Metabolomic Profiling

The dried OCE was reconstituted in a hydroethanolic solution (50:50, *v*/*v*) to improve the extraction of metabolites with different polarities and to ensure optimal chromatographic separation and ionization efficiency. Samples were vortex-mixed for 30 s and filtered through 0.45 μm hydrophilic nylon syringe filters (Millex™, 33 mm; Merck KGaA, Darmstadt, Germany). The filtrates were collected in Micronic microtubes (Micronic B.V., Lelystad, The Netherlands).

A pooled quality control (QC) sample was prepared by combining 50 μL aliquots of each filtered extract and thoroughly vortex-mixing the resulting solution. The QC sample was used to monitor instrument stability and analytical reproducibility throughout the LC-MS sequence [35].

Chromatographic separation was performed on a Gemini C18 column (Phenomenex, Torrance, CA, USA) operated at 40 °C with a flow rate of 0.35 mL/min. The injection volume was 5 μL. The mobile phase consisted of solvent A (ultrapure water containing 0.1% formic acid) and solvent B (acetonitrile containing 0.1% formic acid). The gradient program was as follows: 0.0 min, 3% B; 1.0 min, 10% B; 9.0 min, 50% B; 13.0 min, 100% B; 14.5 min, 3% B, followed by re-equilibration, for a total run time of 18 min.

Mass spectrometric analyses were carried out using an Orbitrap IQ-X Tribrid mass spectrometer (Thermo Fisher Scientific, Bremen, Germany) equipped with an electrospray ionization (ESI) source operating in negative ion mode. Instrumental parameters were set as follows: spray voltage, 2.5 kV; vaporizer temperature, 300 °C; ion transfer tube temperature, 300 °C. Full-scan MS spectra (MS^1^) were acquired over an *m*/*z* range of 70–1050 at a resolving power of 90,000 using the EASY-IC internal calibration system. Data-dependent acquisition (DDA) MS/MS spectra were acquired at a resolving power of 30,000 using high-energy collision dissociation (HCD) with a normalized collision energy of 25% and a maximum injection time of 54 ms.

The analytical sequence included solvent blanks at the beginning and end of each batch, while pooled QC samples were injected every 6–10 analyses to evaluate system stability. Data quality was assessed by monitoring retention time drift (±0.2 min) and signal reproducibility of representative metabolites (coefficient of variation, CV < 15%).

Metabolite annotation was based on accurate mass measurements, retention times (RT), and MS/MS fragmentation patterns. Putative compound identification was achieved through comparison with the METLIN, FooDB, and PubChem databases, together with previously published literature. Annotation confidence was assessed based on RT, accurate mass, MS/MS matching, and spectral similarity scores. Metabolites were classified as putatively identified (MSI Level 2). The most abundant metabolites were selected by the mean relative chromatographic peak area across all analytical replicates. Peak area values were log10-transformed prior to statistical analysis to stabilize variance and are therefore reported as relative abundances. All LC-MS analyses were performed at the Bordeaux Metabolome Platform (MetaboHUB, Bordeaux, France).

### 2.6. DPPH Radical Scavenging Assay

The free radical scavenging activity of OCE was evaluated using the 2,2-diphenyl-1-picrylhydrazyl (DPPH) assay, as previously described by Moumou et al., with minor modifications [17]. Extract solutions were prepared at concentrations ranging from 0.015 to 0.5 mg/mL. Briefly, 100 μL of each extract solution was mixed with 2.9 mL of a methanolic DPPH solution (6 × 10^−5^ M). After incubation in the dark at room temperature for 15 min, the absorbance was measured at 517 nm using a microplate reader (BioTek Gen5, BioTek Instruments Inc., Winooski, VT, USA). Distilled water was used as the negative control, whereas ascorbic acid served as the positive control. Radical scavenging activity was calculated according to the following Equation (2):

(2) $$\mathrm{DPPH}\;\mathrm{inhibition}\;\left(\%\right)=\frac{A_{control}-A_{sample}}{A_{control}}\;\times \;100$$

The antioxidant activity was expressed as the IC_50_ value, defined as the extract concentration required to inhibit 50% of DPPH radicals.

### 2.7. Phosphomolybdenum Assay

Total antioxidant capacity (TAC) was determined using the phosphomolybdenum assay, as previously described by Hadini et al. [36]. Briefly, 50 μL of OCE (1 mg/mL) was mixed with 500 μL of phosphomolybdenum reagent containing 0.6 M sulfuric acid, 28 mM sodium phosphate (NaH_2_PO_4_), and 4 mM ammonium molybdate. The reaction mixture was incubated in a water bath at 95 °C for 90 min and then cooled to room temperature. Absorbance was measured at 695 nm using a microplate reader (BioTek Gen5, BioTek Instruments Inc., Winooski, VT, USA). Results were expressed as milligrams of α-tocopherol equivalents per gram of dry matter (mg α-TocE/g DM).

### 2.8. Evaluation of the Hypolipidemic Effect of OCE In Vivo

The high-fat, high-cholesterol diet (HFCD) was prepared according to the method described by Harnafi et al. [39] and consisted of 81.8% standard laboratory diet supplemented with 16% fat, 2% cholesterol, and 0.2% cholic acid.

Male Swiss albino mice (10 weeks old, 24–30 g) were obtained from the animal facility of the Faculty of Sciences, Mohammed First University (Oujda, Morocco). Animals were housed under controlled environmental conditions (24 ± 2 °C; 12 h light/12 h dark cycle) with free access to food and water. All animal procedures were approved by the Institutional Ethics Committee of Mohammed First University (Ethical Approval No. 044/2024) and carried out in accordance with Directive 2010/63/EU on the protection of animals used for scientific purposes. During the study, all measures were taken to minimize animal suffering and to ensure animal welfare.

Following one week of acclimatization, mice were randomly assigned to four experimental groups (*n* = 8 per group):

- Normolipidemic control (NLC): standard diet + distilled water (0.5 mL/day, oral gavage);
- Hyperlipidemic control (HLC): HFCD + distilled water (0.5 mL/day, oral gavage);
- OCE-treated group (OCE): HFCD + OCE (200 mg/kg body weight/day, administered orally by gavage once daily);
- Simvastatin-treated group (SVT): HFCD + simvastatin (15 mg/kg body weight/day, given orally by gavage once daily).

Treatments were administered daily for 9 weeks.

At the end of the experimental period, mice were fasted overnight (12 h) before blood collection from the retro-orbital sinus under light diethyl ether anesthesia into tubes containing 3.8% trisodium citrate as an anticoagulant. Plasma was separated by centrifugation at 5000 rpm for 5 min and stored for subsequent biochemical analyses. Animals were then euthanized, and the liver, heart, and kidneys were excised, weighed, and stored at −20 °C until analysis.

### 2.9. Biochemical Analysis

#### 2.9.1. Plasma Lipid Profile

Plasma concentrations of TC, TG, LDL-C, and glucose were determined using commercial enzymatic assay kits (BioSystems S.A., Barcelona, Spain), according to the manufacturer’s instructions.

#### 2.9.2. Atherogenic Index and *LDL-C*/*HDL-C* Ratio

The atherogenic index (*AI*) and *LDL-C*/*HDL-C* ratio were calculated using the following Equations (3) and (4):

(3) $$AI=\frac{TC-HDL-C}{HDL-C}$$

(4) $$\mathrm{LDL-C}/\mathrm{HDL-C}=\frac{LDL-C}{HDL-C}$$

#### 2.9.3. Hepatic Lipid Extraction and Quantification

Hepatic lipids were extracted according to the method previously described by Harnafi et al. Briefly, 1 g of liver tissue was homogenized in cold isopropanol (1:10, *w*/*v*) and incubated at 4 °C for 24 h. The homogenate was centrifuged at 2500 rpm for 5 min, and the resulting supernatant was collected.

Aliquots (10 μL) of the extract were mixed with 1 mL of enzymatic reagent for the determination of total cholesterol or triglycerides (BioSystems S.A., Barcelona, Spain), incubated at 37 °C for 10 min, and the absorbance was measured at 500 nm using a UV–Vis Varioskan LUX multimode microplate reader spectrophotometer (Thermo Fisher Scientific, Waltham, MA, USA). Hepatic lipid concentrations were expressed as mg/g wet liver tissue.

#### 2.9.4. Determination of Hepatic Malondialdehyde (MDA)

Lipid peroxidation was evaluated by measuring hepatic malondialdehyde (MDA) levels using the thiobarbituric acid reactive substances (TBARS) assay, as previously described by Ali et al. [40]. Briefly, the TBARS reagent was freshly prepared by dissolving trichloroacetic acid (15%, *w*/*v*) and thiobarbituric acid (0.37%, *w*/*v*) in distilled water, followed by the addition of hydrochloric acid to obtain a final concentration of 0.25 N. The solution was gently heated (approximately 70 °C) until complete dissolution.

For the assay, 0.5 mL of liver homogenate was mixed with 0.5 mL of distilled water and 2 mL of the TBARS reagent. The reaction mixture was heated at 95 °C for 15 min in a boiling water bath, rapidly cooled to room temperature, and centrifuged at 5000 rpm for 10 min. The absorbance of the supernatant was measured at 535 nm using a UV–Vis spectrophotometer (Thermo Fisher Scientific, MA, USA). MDA concentrations were calculated using the molar extinction coefficient of 1.56 × 10^5^ M^−1^ cm^−1^ according to the Beer–Lambert equation and expressed as nmol/mg protein after normalization to protein concentration determined by the Bradford assay [41].

### 2.10. Acute Oral Toxicity Evaluation

Acute oral toxicity of OCE was evaluated according to the OECD Guideline 420 (Fixed Dose Procedure) [42]. Following a one-week acclimatization period, twelve Swiss albino mice were randomly divided into two groups of six animals each: a control group and an OCE-treated group. Each group included three males and three females.

After an overnight fast of 12 h, the control group received distilled water by oral gavage, whereas the treated group received a single oral dose of OCE at 2000 mg/kg body weight. In accordance with OECD Guideline 420, two animals were initially treated, and, in the absence of mortality or evident signs of toxicity after the initial observation period, the remaining animals received the same dose.

Animals were monitored during the first hours after administration and then daily for 14 days for clinical signs of toxicity, including changes in behavior, locomotor activity, respiration, body weight, food intake, ataxia, apathy, and mortality.

### 2.11. Histopathological Examination

Liver and kidney tissues were collected immediately after euthanasia and fixed in 10% neutral-buffered formalin for 72 h [41]. Fixed tissues were dehydrated through a graded ethanol series (30%, 50%, 70%, 90%, and 100%), cleared in toluene, embedded in paraffin wax, and sectioned at 5 μm using a rotary microtome.

Sections were mounted on gelatin-coated glass slides, dried at 45 °C for 1 h, deparaffinized in toluene, and rehydrated through descending ethanol concentrations (100%, 95%, and 70%) to distilled water. Histological staining was performed with hematoxylin for 5 min, followed by differentiation in acid alcohol, bluing in ammoniacal alcohol, counterstaining with eosin for 4 min, dehydration through graded ethanol solutions, clearing in toluene, and permanent mounting.

Histopathological evaluation was performed using a light microscope (Olympus CX21, Olympus Corporation, Tokyo, Japan), and representative micrographs were digitally acquired.

### 2.12. Molecular Docking Simulations and ADMET Analysis

The three-dimensional structures of the ligands, namely piscidic acid, caffeic acid, *p*-coumaric acid, isorhamnetin-3-*O*-rutinoside, quercetin-3-*O*-rutinoside (Rutin), dihydrokaempferol, isorhamnetin, quercetin, naringenin, kaempferol, and simvastatin, were retrieved from the PubChem database. Ligands were imported into PyRx v0.8, converted into PDBQT format, and energy-minimized using the Universal Force Field (UFF) to obtain stable low-energy conformations prior to docking. The crystal structures of twelve proteins involved in lipid metabolism and cholesterol homeostasis were downloaded from the RCSB Protein Data Bank (PDB): PPARα (PDB ID: 1I7G), NPC1L1 (7DF8), HMG-CoA reductase (1HWK), ABCA1 (5XJY), CYP7A1 (3DAX), CETP (2OBD), LXRα (2ACL), ACAT (6L47), FXR (1OSV), FAS (1XKT), ACLY (3MWD), and PCSK9 (6U26). Protein preparation was performed using BIOVIA Discovery Studio 2025 SP1 (Dassault Systèmes BIOVIA, San Diego, CA, USA) by removing water molecules, co-crystallized ligands, and heteroatoms, followed by the addition of polar hydrogen atoms.

Molecular docking simulations were performed in PyRx v0.8 using AutoDock Vina v1.2.x as the docking engine. Blind docking was applied to explore the entire protein surface without predefined binding pockets. Docking exhaustiveness was set to 8, providing an appropriate balance between computational accuracy and calculation time. Binding affinities were expressed as binding free energies (ΔG, kcal/mol), and, for each ligand–protein complex, the pose showing the lowest ΔG value was selected for further analysis. Protein–ligand interactions, including hydrogen bonds, hydrophobic contacts, and electrostatic interactions, were analyzed and visualized in both two- and three-dimensional representations using BIOVIA Discovery Studio [17].

The pharmacokinetic and toxicological properties of the identified *Opuntia* phytochemicals were predicted using in silico tools. Molecular structures were converted into canonical SMILES format prior to analysis. ADME properties, including human intestinal absorption (HIA), Caco-2 permeability, blood–brain barrier (BBB) penetration, and interactions with CYP3A4 and CYP2D6 enzymes, were predicted using ADMET-AI (v1.3.1). Toxicological endpoints, including oral LD_50_, organ toxicity, mutagenicity, and carcinogenicity, were evaluated using ProTox 3.0. Drug-likeness was assessed by comparison with compounds available in the DrugBank database, using simvastatin as the reference drug [43].

### 2.13. Statistical Analysis

Statistical analyses were performed using GraphPad Prism v9.5.0 (GraphPad Software, San Diego, CA, USA), whereas multivariate analyses and graphical visualizations were carried out using RStudio v4.3.2 (R Foundation for Statistical Computing, Vienna, Austria). All in vitro phytochemical assays were performed in triplicate, and results are presented as mean ± standard deviation (SD).

Data normality was assessed using the Shapiro–Wilk test, whereas homogeneity of variances was evaluated using Levene’s and Bartlett’s tests. Differences among groups were analyzed by one-way analysis of variance (ANOVA) followed by Tukey’s multiple comparison test. Pearson correlation coefficients were calculated and visualized as heatmaps to investigate relationships among phytochemicals, metabolites, antioxidant parameters, and molecular docking results. Linear regression analysis was applied to evaluate the association between molecular descriptors and docking binding affinities. Scatter plots with 95% confidence intervals were generated using the ggplot2 package in R. Statistical significance was set at *p* < 0.05.

## 3. Results

An integrative workflow combining phytochemical characterization, in vitro antioxidant assays, in vivo evaluation, untargeted metabolomics, and in silico analyses was applied to investigate the hypolipidemic potential of *O. ficus-indica* cladode extract (OCE). The combined approach provided complementary evidence supporting the biological activity of the extract and offered mechanistic insights into its effects on lipid metabolism.

### 3.1. In Vitro Phytochemical Characterization

#### 3.1.1. Total Phenolic, Flavonoid, and Condensed Tannin Contents

Liquid–liquid partitioning of the crude extract yielded a predominantly polar composition, with recovery yields of approximately 75% for the aqueous fraction (AQFr) and 15% for the ethyl acetate fraction (EAFr).

The crude extract (OCE) exhibited the highest concentrations of total phenolics (316.56 ± 0.65 mg GAE/g DM), total flavonoids (33.36 ± 1.03 mg RE/g DM), and condensed tannins (47.67 ± 2.84 mg CE/g DM) (Table 1). Partitioning markedly reduced the concentration of these phytochemicals in both fractions. The AQFr contained a significantly higher total phenolic content than the EAFr (159.01 vs. 120.81 mg GAE/g DM; *p* < 0.05), whereas flavonoids were significantly enriched in the EAFr (16.28 ± 0.70 mg RE/g DM) compared with the AQFr (10.49 ± 1.11 mg RE/g DM). In contrast, condensed tannins were similarly distributed between the two fractions, with no significant differences detected.

#### 3.1.2. Antioxidant Activity of OCE and Its Fractions

**Figure 1:**
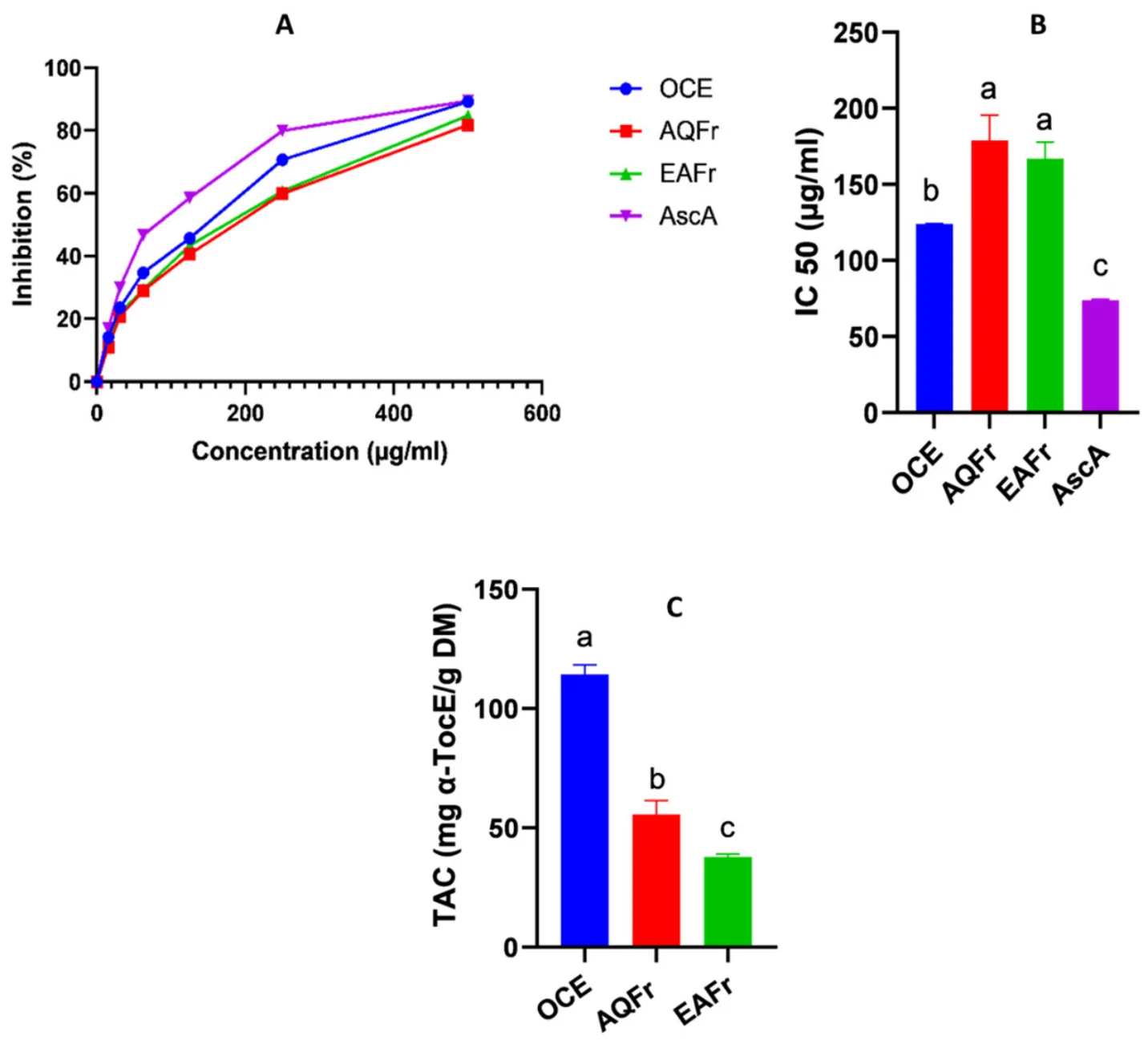
Antioxidant activity of the crude extract (OCE) and its fractions (AQFr and EAFr). (**A**) DPPH radical scavenging curves. (**B**) IC_50_ values obtained from the DPPH assay. (**C**) Total antioxidant capacity (TAC) expressed as mg α-tocopherol equivalents (α-TocE)/g DM. Different letters indicate statistically significant differences (*p* < 0.05; one-way ANOVA followed by Tukey’s post hoc test). OCE, *Opuntia* cladode extract; AQFr, aqueous fraction; EAFr, ethyl acetate fraction; AscA, ascorbic acid.

The antioxidant activity of OCE and its fractions was evaluated using the DPPH radical scavenging assay and the phosphomolybdenum total antioxidant capacity (TAC) assay (Figure 1). As expected, ascorbic acid showed the highest radical scavenging activity (IC_50_ = 73.87 ± 0.57 μg/mL). Among the *Opuntia* preparations, the crude extract displayed the strongest antioxidant activity, with a significantly lower IC_50_ value (124.26 ± 0.32 μg/mL) than both the EAFr (166.94 ± 10.81 μg/mL) and AQFr (178.80 ± 16.68 μg/mL) (*p* < 0.05) (Figure 1A,B). A similar trend was observed in the TAC assay (Figure 1C). The crude extract exhibited the highest total antioxidant capacity (114.25 ± 4.02 mg α-TocE/g DM), followed by the AQFr (55.71 ± 5.72 mg α-TocE/g DM) and the EAFr (37.85 ± 1.20 mg α-TocE/g DM), with all differences being statistically significant (*p* < 0.05).

#### 3.1.3. UHPLC–Orbitrap IQ-X Tribrid MS/MS Metabolomic Profiling of OCE

Untargeted UHPLC–Orbitrap IQ-X Tribrid MS/MS analysis enabled the tentative identification of 102 metabolites, revealing the broad chemical diversity of *O. ficus-indica* cladode extract (Table 2). The metabolome was dominated by phenolic acids, flavonoids, organic acids, amino acids, sugars, and lipid derivatives, indicating the presence of diverse primary and secondary metabolites. Among the 22 annotated phenolic acids and related compounds, piscidic acid (C_11_H_12_O_7_) was the most abundant compound (peak area: 1.32 × 10^9^), supporting its prominence among the phenolic constituents detected in *Opuntia* cladodes (Table S1). Other major phenolic constituents included caffeic acid, *p*-coumaric acid, ferulic acid derivatives, feruloyl quinic acids, salicylic acid derivatives, and glucosylated hydroxycinnamates. The flavonoid fraction comprised 30 compounds, predominantly represented by glycosylated derivatives of isorhamnetin, quercetin, and kaempferol, including rutinosides, glucosides, galactosides, and vicianosides. Other flavonoids, such as xanthorhamnin, quercimeritrin, naringenin, taxifolin derivatives, and acacetin, further contributed to the phytochemical diversity of the extract. Primary metabolism was mainly represented by abundant organic acids, including citric, galactaric, gluconic, malic, quinic, and aconitic acids, together with carbohydrates and polyols such as galactose, mannitol, arabitol, lyxose, raffinose, and stachyose. Additional metabolites included amino acids, hydroxylated fatty acids, phospholipids, lignans, nucleosides, vitamins, and other specialized metabolites, further illustrating the chemical diversity of the extract.

**Figure 2:**
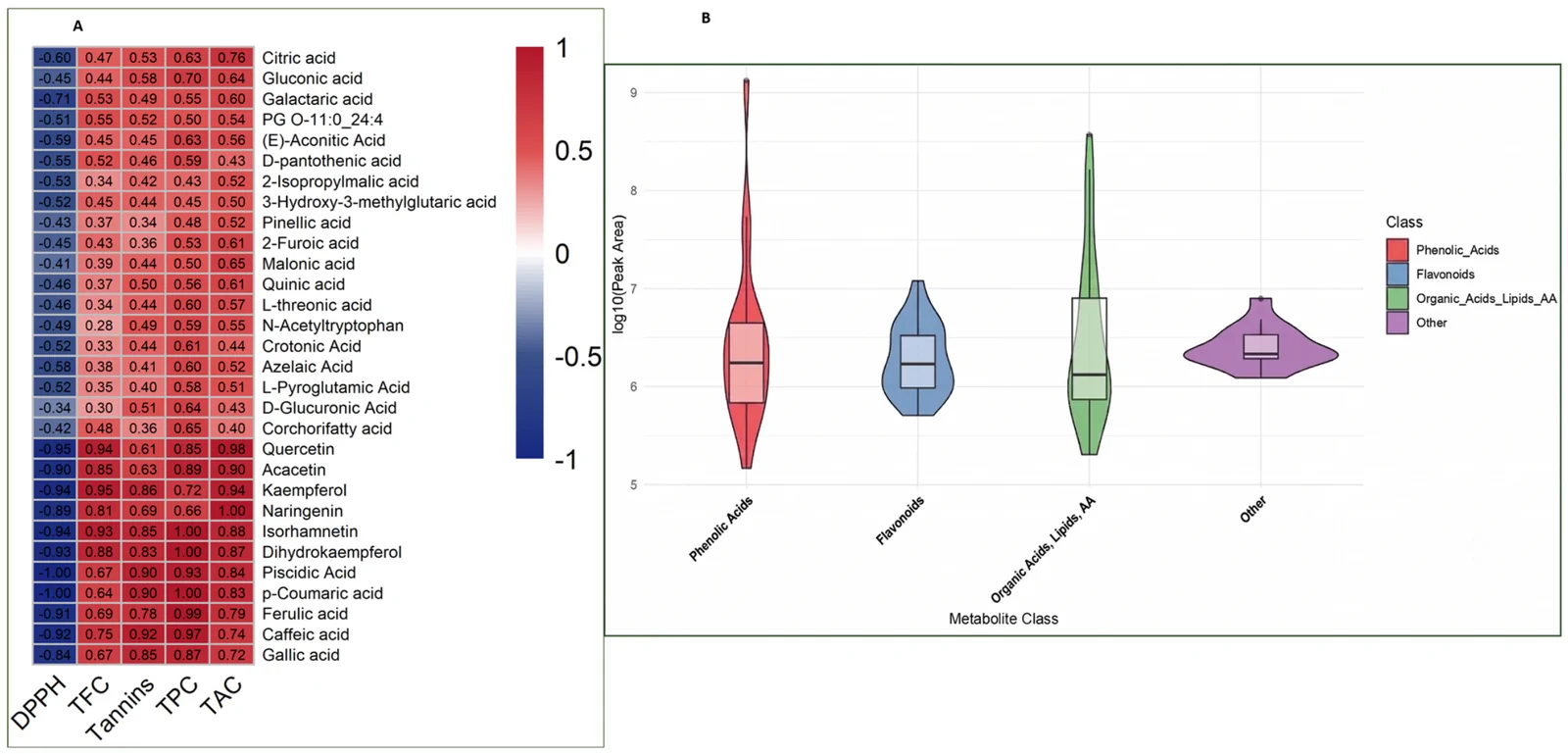
(**A**) Pearson correlation heatmap showing the relationships among the 30 most abundant metabolites, antioxidant activities (DPPH and TAC), and phytochemical parameters (TPC, TFC, and condensed tannins). The values and color scale represent Pearson correlation coefficients. DPPH, 2,2-diphenyl-1-picrylhydrazyl; TAC, total antioxidant capacity; TPC, total phenolic content; TFC, total flavonoid content. (**B**) Distribution of metabolites according to chemical class and relative abundance (log_10_ peak area).

Correlation analysis further supported the association between metabolite composition and antioxidant activity (Figure 2A). Strong positive correlations (r > 0.85) were observed between total phenolic content (TPC), total antioxidant capacity (TAC), and major phenolic constituents, including piscidic acid, isorhamnetin, quercetin, and naringenin. Conversely, these metabolites showed strong negative correlations with DPPH IC_50_ values (r ≈ −1), indicating that higher metabolite abundances were associated with stronger radical-scavenging activity. Organic acids and lipid-derived metabolites generally displayed weaker correlations with antioxidant parameters, indicating weaker associations with the measured antioxidant activity. The abundance distribution of the identified metabolites is presented in Figure 2B. Phenolic acids exhibited the widest dynamic range, largely driven by the exceptionally high abundance of piscidic acid, whereas flavonoids showed a narrower abundance distribution despite their greater structural diversity. Organic acids represented another highly abundant metabolite class, highlighting the coexistence of abundant primary and secondary metabolites in the extract.

### 3.2. Metabolic Effects of OCE in HFD-Fed Mice

#### 3.2.1. Body Weight Gain and Organ Weights

Following the 9-week treatment period, body weight gain and organ weights were evaluated to assess the metabolic effects of OCE (Table 3). Mice fed the high-fat diet (HLC) exhibited a significantly greater body weight gain than the normal control (NLC) group (*p* < 0.05). OCE supplementation markedly attenuated weight gain, reducing ΔBWG to levels comparable to those observed in the simvastatin-treated group (SVTG), with no significant differences between these two treatments. A significant increase in liver weight was observed in HLC mice compared with NLC animals, which was consistent with hepatic lipid accumulation induced by the high-fat diet. In contrast, OCE treatment significantly attenuated this increase, resulting in liver weights comparable to those of the NLC and SVTG groups. Heart weight did not differ significantly among the experimental groups. Kidney weight remained unchanged following OCE administration, whereas a slight but significant increase was observed in the SVTG compared with the HLC and OCE-treated groups.

#### 3.2.2. Plasma Lipid Profile, Glycemic Status, Atherogenic Indices, and Hepatic Lipid Content

**Figure 3:**
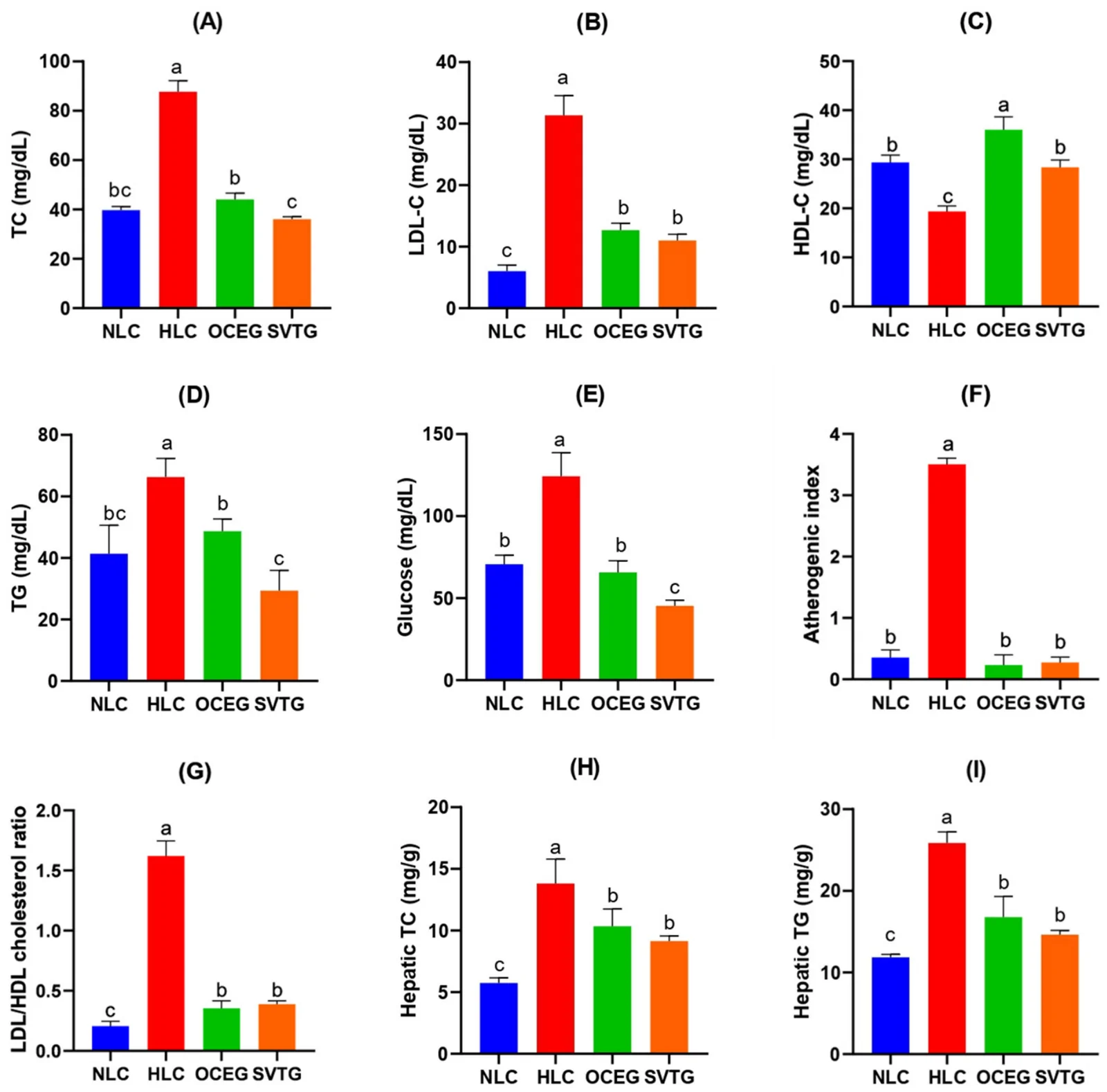
Effects of *Opuntia* cladode extract (OCE) on plasma and hepatic biochemical parameters. (**A**) TC; (**B**) LDL-C; (**C**) HDL-C; (**D**) TG; (**E**) glucose; (**F**) atherogenic index; (**G**) LDL-C/HDL-C ratio; (**H**) hepatic TC; and (**I**) hepatic TG. Values are expressed as mean ± SD (*n* = 8). Different letters indicate statistically significant differences (*p* < 0.001; one-way ANOVA followed by Tukey’s post hoc test). NLC, normal control; HLC, hyperlipidemic control; OCEG, *Opuntia* cladode extract-treated group; SVTG, simvastatin-treated group.

The effects of OCE on plasma biochemical parameters and hepatic lipid accumulation after 9 weeks of HFD feeding are presented in Figure 3A–I. As expected, the HLC group exhibited marked metabolic alterations compared with the NLC group, confirming the successful induction of hyperlipidemia (*p* < 0.001). Compared with HLC mice, OCE administration significantly reduced plasma TC, LDL-C, TG, and glucose levels (Figure 3A–E). The lipid-lowering effect of OCE was comparable to that of simvastatin for TC and LDL-C, whereas HDL-C levels remained significantly higher in the OCE-treated group than in the SVTG. Moreover, OCE nearly normalized fasting glucose concentrations, which were not significantly different from those of the NLC group. Cardiovascular risk indices showed a similar trend (Figure 3F,G). Both the atherogenic index (AI) and the LDL-C/HDL-C ratio were markedly increased in HLC mice and were significantly reduced following OCE treatment (*p* < 0.001). The LDL-C/HDL-C ratio in the OCE-treated group was comparable to that observed in the simvastatin group, indicating an improvement in the plasma lipoprotein profile. The HFD also induced a significant accumulation of hepatic lipids (Figure 3H,I). OCE administration significantly decreased both hepatic TC and TG contents compared with the HLC group, with values approaching those observed in the simvastatin-treated group.

#### 3.2.3. Hepatic Malondialdehyde Levels

**Figure 4:**
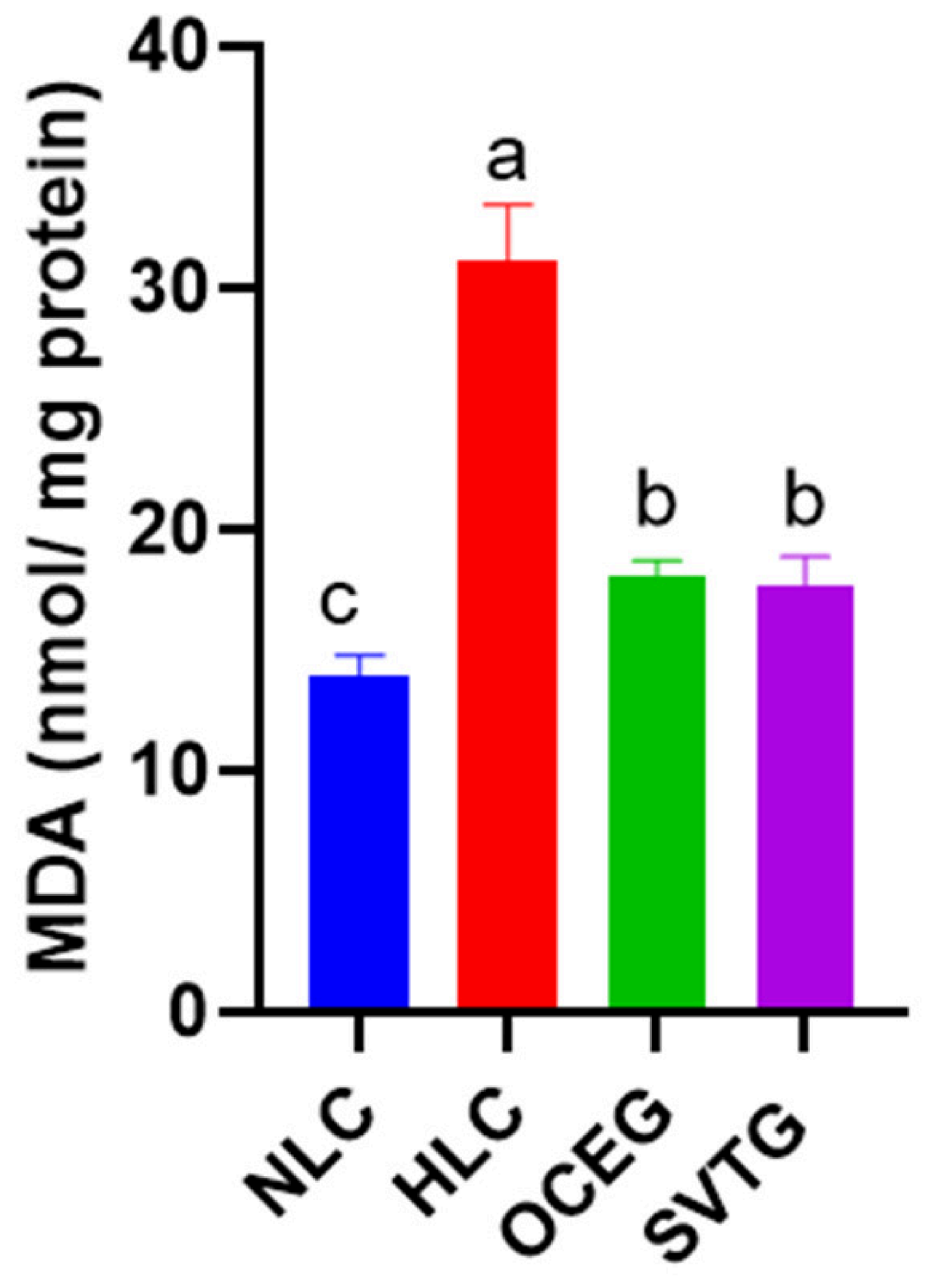
Effect of OCE and simvastatin on hepatic MDA levels in mice. Values are expressed as mean ± SD (*n* = 8). Different letters indicate statistically significant differences (*p* < 0.001; one-way ANOVA followed by Tukey’s post hoc test). NLC, normal control; HLC, hyperlipidemic control; OCEG, *Opuntia* cladode extract-treated group; SVTG, simvastatin-treated group.

Hepatic malondialdehyde (MDA) levels were measured as an indicator of lipid peroxidation and oxidative stress (Figure 4). HFD feeding significantly increased MDA levels in the HLC group compared with the NLC group (*p* < 0.001), indicating increased hepatic lipid peroxidation. OCE treatment significantly reduced hepatic MDA levels compared with the HLC group, with values comparable to those observed in the simvastatin-treated group. These results indicate that OCE reduced HFD-associated hepatic lipid peroxidation.

#### 3.2.4. Histopathological Evaluation of Hepatic and Renal Tissues

**Figure 5:**
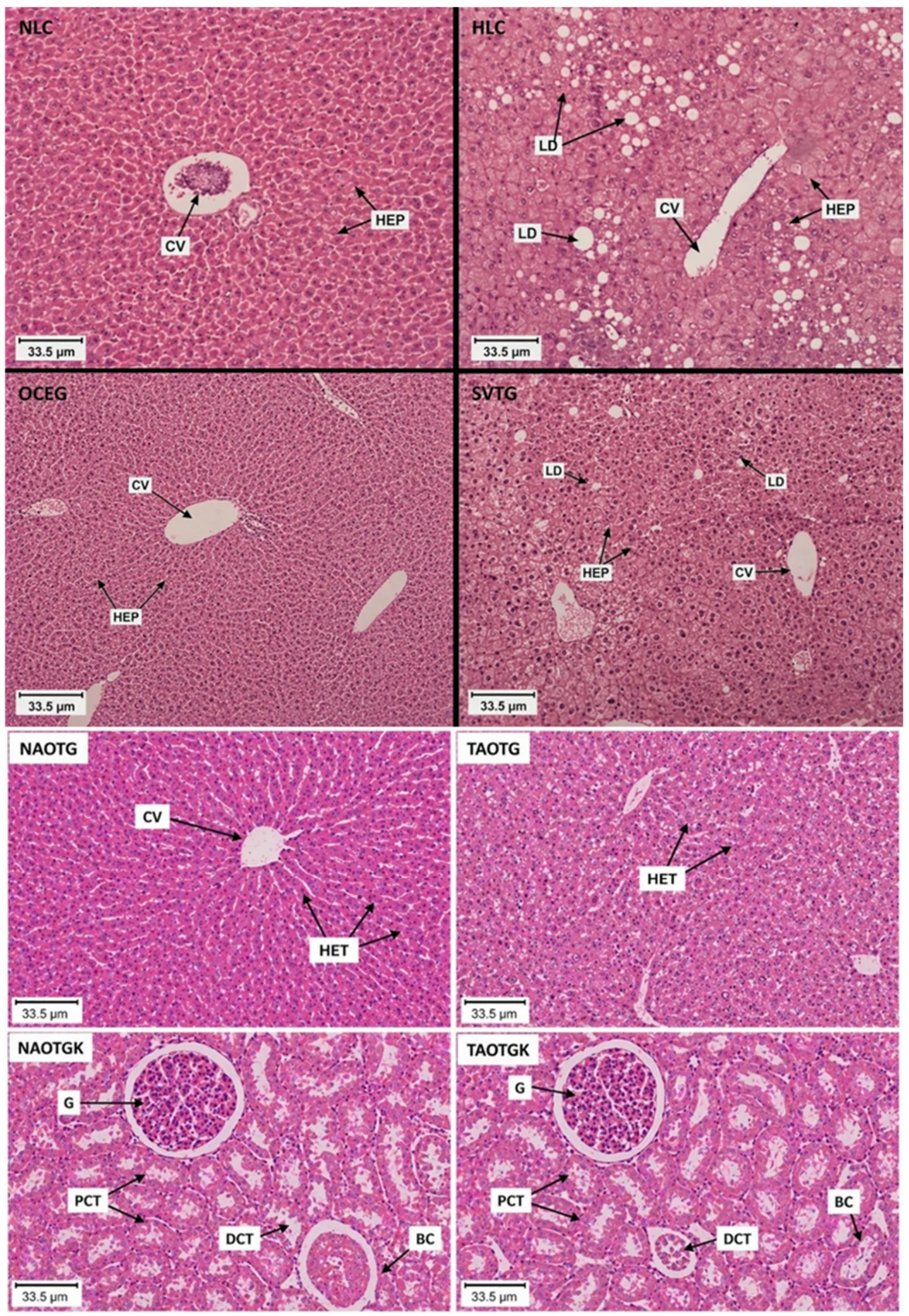
Histopathological evaluation of liver and kidney tissues following OCE treatment. Upper panels show liver sections from the 9-week HFD experiment: NLC, normal lipid control; HLC, hyperlipidemic control, showing lipid droplet accumulation; OCEG, *Opuntia* cladode extract-treated group; SVTG, simvastatin-treated group. Lower panels show liver and kidney sections from the 14-day acute oral toxicity study after administration of OCE at 2000 mg/kg: NAOTG, acute toxicity control liver; TAOTG, OCE-treated acute toxicity liver; NAOTGK, acute toxicity control kidney; TAOTGK, OCE-treated acute toxicity kidney. CV, central vein; HEP/HET, hepatocytes; LD, lipid droplets; G, glomerulus; BC, Bowman’s capsule; PCT, proximal convoluted tubule; DCT, distal convoluted tubule. Hematoxylin and eosin (H&E) staining. Scale bar: 33.5 μm.

Histopathological examination of liver sections revealed marked differences among the experimental groups after 9 weeks of treatment (Figure 5). The NLC group showed normal hepatic architecture, with well-preserved hepatocytes and regularly organized hepatic cords around the central vein. In contrast, the HLC group exhibited pronounced hepatic steatosis, characterized by the accumulation of large lipid droplets within hepatocytes, indicating substantial hepatic lipid accumulation associated with HFD feeding.

Simvastatin treatment reduced hepatic lipid accumulation while preserving the overall lobular architecture, although residual cytoplasmic lipid vacuoles were still observed. Similarly, OCE administration markedly improved hepatic morphology, with a substantial reduction in steatotic vacuoles and a restoration of the overall tissue architecture toward that observed in the NLC group. Hepatocytes appeared regularly organized, and hepatic cords were clearly arranged around the central vein. Histological examination performed after the 14-day acute oral toxicity study showed no evident tissue damage in the liver or kidneys of mice treated with OCE at 2000 mg/kg (Figure 5). Liver sections from both control and treated animals displayed preserved parenchymal organization, normal hepatocyte morphology, and no signs of steatosis, inflammation, or necrosis. Similarly, kidney sections showed intact renal architecture, with normal glomeruli, Bowman’s capsules, and proximal and distal convoluted tubules, without evidence of epithelial damage or tubular obstruction.

### 3.3. Acute Oral Toxicity Assessment

The acute oral toxicity of OCE was evaluated after administration of a single oral limit dose of 2000 mg/kg body weight. No mortality or treatment-related clinical signs were observed in the OCE-treated group during the 14-day observation period. In addition, no relevant changes in behavior, locomotor activity, respiration, or autonomic responses were detected compared with the control group.

Based on these findings, the acute oral LD_50_ of OCE was estimated to be greater than 2000 mg/kg body weight. According to the Globally Harmonized System (GHS) classification criteria, OCE showed low acute oral toxicity under the experimental conditions used.

These results indicate a favorable preliminary safety profile of OCE following single-dose oral administration.

### 3.4. Molecular Docking Analysis of OCE Metabolites Against Key Targets Involved in Lipid Metabolism

**Figure 6:**
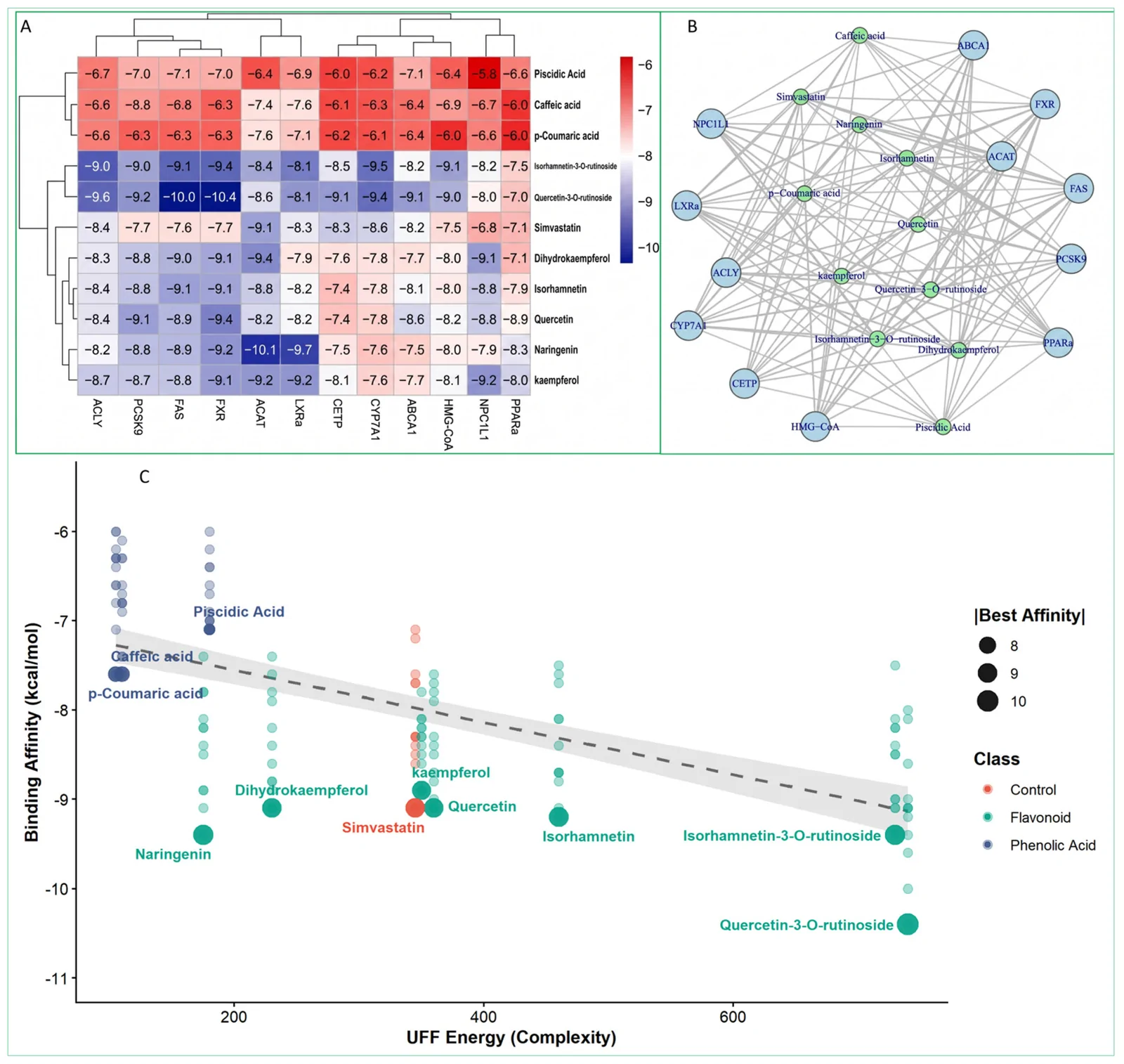
Integrated molecular docking analysis of *O. ficus-indica* phytochemicals against key proteins involved in lipid metabolism. (**A**) Heatmap of binding free energies (ΔG), with hierarchical clustering based on similarity in binding-energy profiles; darker blue indicates more negative binding energies; (**B**) ligand–target interaction network with gray lines indicating predicted ligand–target interactions; and (**C**) relationship between molecular complexity (UFF energy) and binding affinity. Point size represents best affinity. The dashed line represents the fitted linear regression, and the gray shaded area indicates the 95% confidence interval. ABCA1, ATP-binding cassette transporter A1; ACAT, acyl-CoA cholesterol acyltransferase; CETP, cholesteryl ester transfer protein; CYP7A1, cholesterol 7α-hydroxylase; FAS, fatty acid synthase; FXR, farnesoid X receptor; HMG-CoA, 3-hydroxy-3-methylglutaryl-coenzyme A; LXRa, liver X receptor alpha; NPC1L1, Niemann-Pick C1-like 1; PCSK9, proprotein convertase subtilisin/kexin type 9; PPARa, peroxisome proliferator-activated receptor alpha; UFF, Universal Force Field.

**Figure 7:**
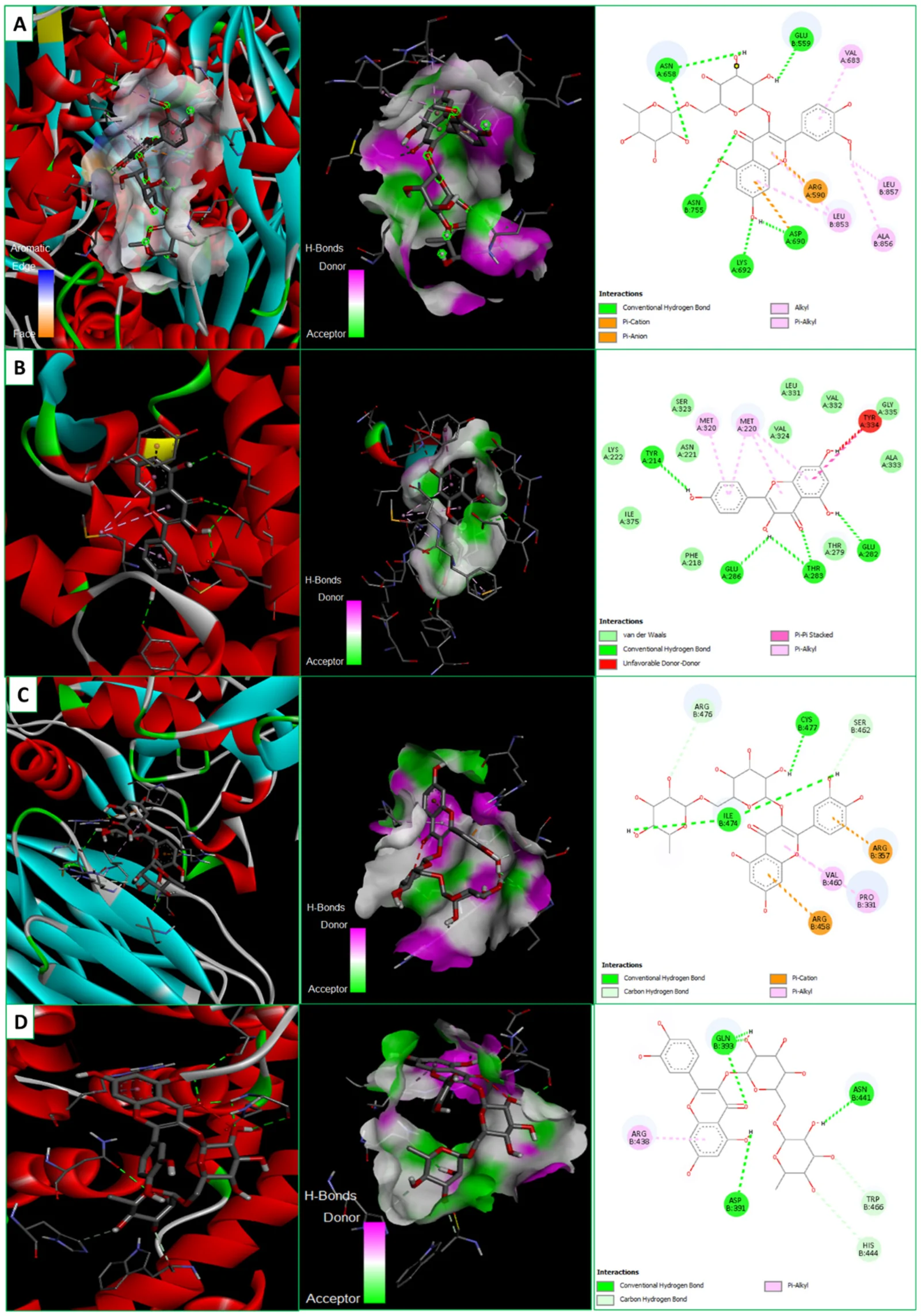
Representative molecular docking interactions between major OCE flavonoids and lipid metabolism-related proteins. (**A**) Isorhamnetin-3-*O*-rutinoside/HMG-CoA reductase; (**B**) Kaempferol/PPARα; (**C**) Quercetin-3-*O*-rutinoside/PCSK9; (**D**) Quercetin-3-*O*-rutinoside/FXR. Left: 3D ribbon representations showing α-helices (red), β-sheets (cyan), and loops (green). Middle: Binding surfaces showing hydrogen-bond donors (magenta) and acceptors (green). Right: 2D interaction diagrams showing conventional hydrogen bonds (green), π–cation/π–anion interactions (orange), alkyl/π–alkyl interactions (pink), van der Waals interactions (light green), and unfavorable clashes (red).

**Figure 8:**
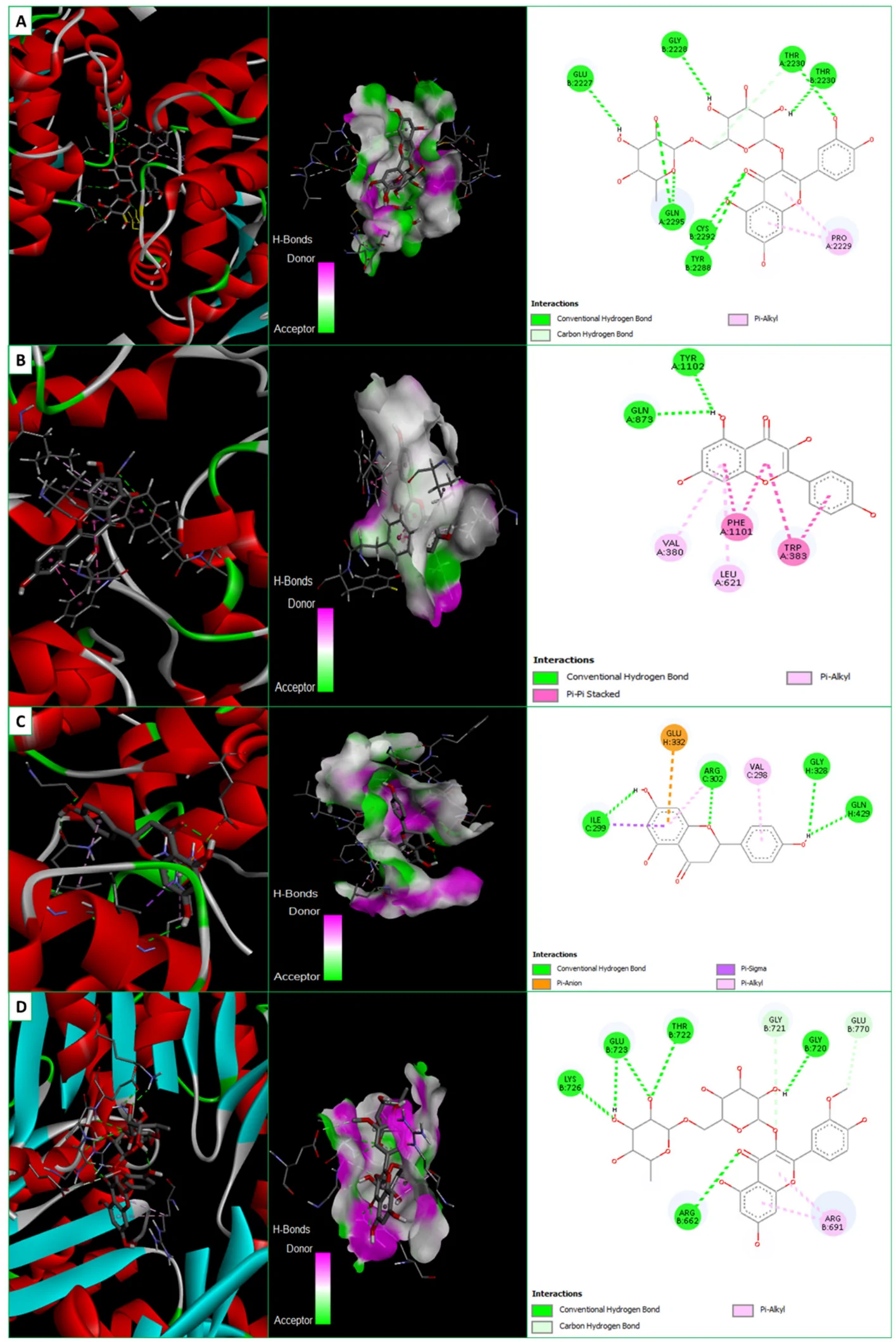
Representative molecular docking interactions between major OCE flavonoids and lipid metabolism-related proteins. (**A**) Quercetin-3-*O*-rutinoside/FAS; (**B**) Kaempferol/NPC1L1; (**C**) Naringenin/LXRα; (**D**) Isorhamnetin-3-*O*-rutinoside/ACLY. Left panels (3D ribbons) show α-helices (red), β-sheets (cyan), and loops (green); middle panels (hydrogen-bond surfaces) show hydrogen-bond donors (magenta) and acceptors (green); right panels (2D diagrams) show chemical structures with oxygen atoms in red and carbon atoms in grey, together with non-covalent interactions, including conventional hydrogen bonds (dark green), carbon–hydrogen bonds (light green), π–anion interactions (orange), π–π stacked interactions (dark pink), π–sigma interactions (purple), and π–alkyl interactions (light pink).

The molecular docking results are summarized in Figure 6, Figure 7 and Figure 8, integrating binding affinity analysis, interaction networks, and structural interaction profiles for eleven ligands (ten *Opuntia*-derived phytochemicals and simvastatin as the reference compound) against twelve proteins involved in cholesterol homeostasis and lipid metabolism.

Docking simulations revealed predicted binding free energies (ΔG) ranging from −5.8 to −10.4 kcal/mol, indicating favorable predicted binding affinities between several OCE metabolites and the selected protein targets (Figure 6A). Among all compounds, the glycosylated flavonoids exhibited the strongest binding affinities. In particular, quercetin-3-*O*-rutinoside showed the lowest ΔG values with FXR (−10.4 kcal/mol) and FAS (−10.0 kcal/mol). Other flavonoids, including naringenin, quercetin, kaempferol, and isorhamnetin derivatives, also displayed favorable predicted binding affinities depending on the target protein. By comparison, simvastatin exhibited ΔG values ranging from −6.8 to −9.1 kcal/mol, whereas phenolic acids such as piscidic, caffeic, and *p*-coumaric acids generally showed more moderate binding energies (approximately −6.0 to −7.5 kcal/mol).

The interaction network (Figure 6B) highlighted the multitarget binding profiles of OCE phytochemicals. Proteins including HMG-CoA reductase, PCSK9, ACAT, and PPARα interacted with multiple ligands, while several metabolites, particularly piscidic acid and hydroxycinnamic acid derivatives, showed interactions with multiple investigated targets. Furthermore, the relationship between molecular complexity, estimated by Universal Force Field (UFF) energy, and docking affinity revealed a positive overall trend (Figure 6C), with flavonoids generally clustering in the region associated with more favorable predicted binding affinities, whereas phenolic acids were distributed within the intermediate affinity range.

Representative two- and three-dimensional docking models are shown in Figure 7 and Figure 8. These analyses revealed numerous hydrogen-bond interactions involving amino acid residues such as GLU, ARG, ASN, ASP, LYS, and THR. For example, isorhamnetin-3-*O*-rutinoside interacted with ASN658 and GLU559 within the HMG-CoA reductase binding site (Figure 7A), whereas quercetin-3-*O*-rutinoside formed hydrogen bonds with GLN333 and ASP331 in FXR (Figure 7D). Hydrophobic interactions, including π–π stacking and π–alkyl contacts, were frequently observed, particularly between flavonoid aromatic rings and residues such as PHE and TRP, as illustrated by the kaempferol–NPC1L1 complex (Figure 8B). Additional interactions, including π-anion, π-cation, π-sigma, and sulfur-mediated contacts, were also observed and may contribute to ligand stabilization within the binding sites.

Overall, the docking analysis revealed that several OCE flavonoids exhibited favorable predicted multitarget binding profiles, with docking affinities comparable or even better than simvastatin in certain targets. These results suggest that these compounds may contribute to the biological activity of OCE and provide hypotheses for further experimental validation of their potential roles in lipid metabolism.

### 3.5. ADMET Prediction of the Major OCE Bioactive Compounds

**Figure 9:**
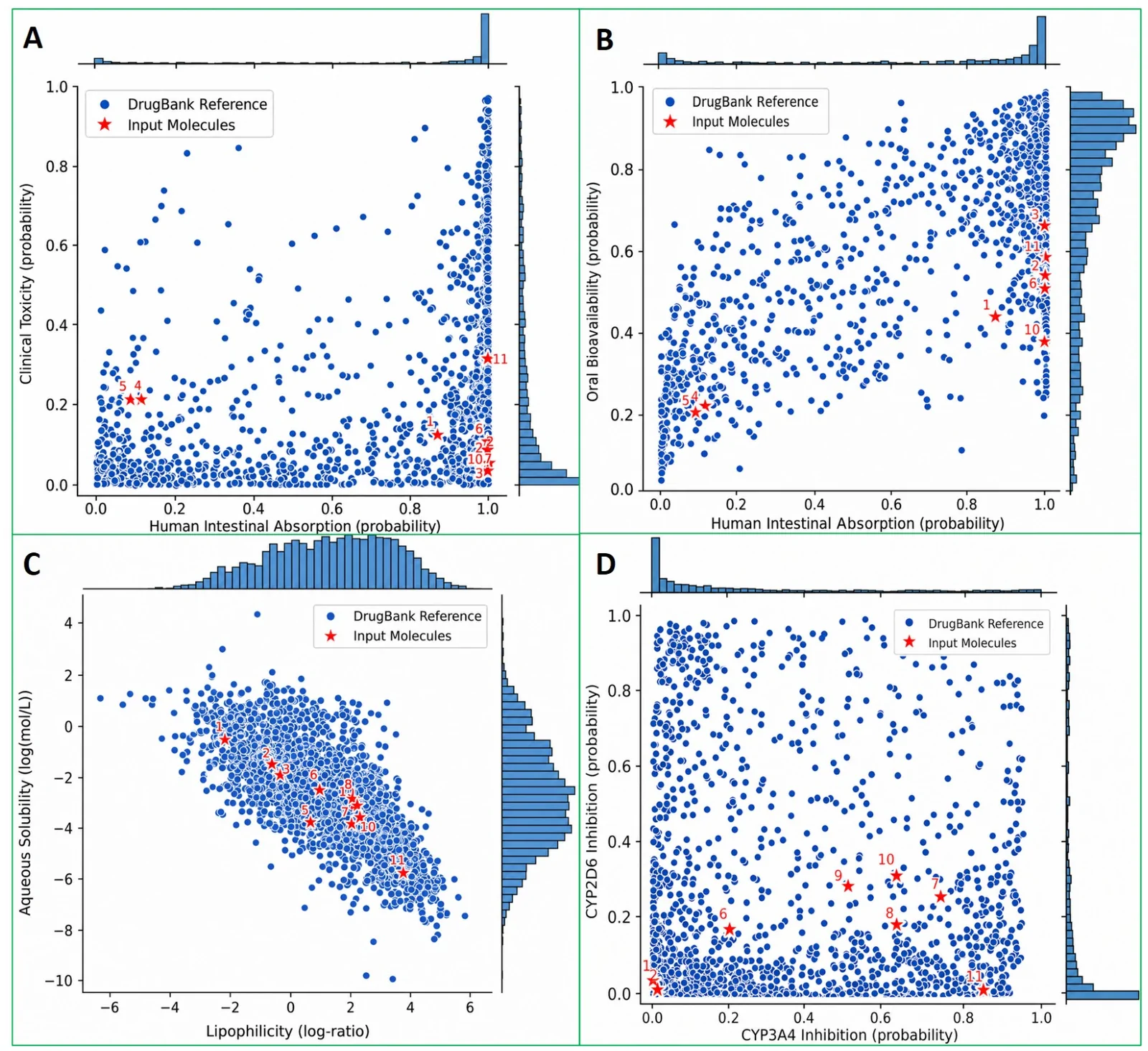
Predicted ADMET profiles of the principal *O. ficus-indica* metabolites generated using ADMET-AI. Compounds: (1) piscidic acid; (2) caffeic acid; (3) *p*-coumaric acid; (4) isorhamnetin-3-*O*-rutinoside; (5) quercetin-3-*O*-rutinoside (rutin); (6) dihydrokaempferol; (7) isorhamnetin; (8) quercetin; (9) naringenin; (10) kaempferol; and (11) simvastatin. (**A**) Human intestinal absorption (HIA) versus clinical toxicity. (**B**) HIA versus oral bioavailability. (**C**) Lipophilicity versus aqueous solubility. (**D**) Predicted CYP3A4 versus CYP2D6 inhibition.

The predicted pharmacokinetic and toxicological properties of the main *O. ficus-indica* phytochemicals were evaluated using ADMET-AI and ProTox 3.0 (Figure 9). Generally, most aglycone flavonoids were predicted to comply with Lipinski’s rule of five and to exhibit favorable predicted intestinal absorption, whereas glycosylated flavonoids showed lower predicted intestinal absorption, likely due to their higher polarity. Compounds were predicted to be inactive as CYP3A4 inhibitors, except for naringenin. Toxicological predictions revealed generally low predicted hepatotoxicity and favorable toxicity profiles. These computational predictions provide complementary information on the predicted pharmacokinetic and toxicological properties of the annotated phytochemicals, although they require experimental validation. The detailed ADMET parameters and toxicity predictions are given in Supplementary Tables S2 and S3.

## 4. Discussion

The present study provides a comprehensive evaluation of the hypolipidemic, antioxidant, and hepatoprotective potential of the aqueous *O. ficus-indica* cladode extract (OCE). By integrating untargeted metabolomics, antioxidant assays, in vivo pharmacological evaluation, and in silico molecular modeling, this study provides complementary evidence supporting the multi-target biological activity of OCE and offers mechanistic insights into its effects on lipid metabolism.

Oral administration of OCE markedly attenuated the metabolic disturbances induced by the HFD, with an overall lipid-lowering effect comparable to that observed with simvastatin under the experimental conditions used. OCE significantly reduced serum TC, TG, and LDL-C while preserving HDL-C levels, which remained significantly higher than those observed in the simvastatin-treated group. Furthermore, hepatic TC and TG contents were reduced, together with reduced hepatic lipid accumulation, suggesting an improvement in lipid dysregulation associated with MASLD/NAFLD [8]. These changes were accompanied by a significant reduction in both the atherogenic index (AI) and the LDL-C/HDL-C (Castelli) ratio, consistent with a more favorable atherogenic lipid profile [44]. OCE also restored fasting glucose concentrations to values comparable with those of the normolipidemic control group, supporting its beneficial effect on glucose homeostasis. In line with previous findings, similar metabolic improvements have been associated with modulation of the IRS-1/PI3K/Akt signaling pathway by dietary phenolic compounds [45]. Collectively, these findings indicate that the hypolipidemic and hepatoprotective effects of OCE may result from the combined contribution of multiple metabolites present in the extract [46,47,48]. The administered OCE dose should also be considered in the context of translational relevance. The dose used in mice (200 mg/kg b.w.) is equivalent to a human equivalent dose (HED) of about 16.2 mg/kg b.w., using body-surface-area (BSA) conversion [49]. This is equivalent to approximately 1.13 g dry extract per day for a 70 kg adult, providing a preliminary estimate of the corresponding human exposure and suggesting that the dose used in the present animal model may be relevant for further nutritional or nutraceutical investigation. However, this conversion does not take into account interspecies differences in absorption, metabolism, bioavailability or tissue exposure and should not be interpreted as a recommended human dose. Additional dose–response and pharmacokinetic studies are required to define the appropriate dose and further assess the translational relevance of OCE in humans.

Phenolic compounds represented an important class of specialized metabolites putatively annotated in OCE. Piscidic acid was tentatively identified as the most abundant detected phenolic constituent and a characteristic biomarker of *Opuntia* cladodes [35,47,48]. Several flavonoids, including isorhamnetin, quercetin, kaempferol, naringenin, and their glycosylated derivatives, were also putatively identified. These metabolites may contribute to lipid homeostasis through multiple complementary molecular pathways rather than through a single pharmacological target, as in the case of simvastatin. Previous studies have shown that phenolic constituents, particularly piscidic acid and isorhamnetin derivatives from *Opuntia*, may downregulate HMGCR expression, thereby limiting endogenous cholesterol biosynthesis and inhibiting intestinal cholesterol absorption across Caco-2 cell monolayers [46,48]. In parallel, enhanced hepatic LDL clearance has been associated with increased LDL receptor (LDLR) expression and reduced PCSK9-mediated receptor degradation [50,51,52]. Likewise, the reduction in circulating TG observed in the present study may reflect decreased hepatic lipogenesis through activation of the AMPK/SREBP-1c pathway, with subsequent inhibition of ACC and FAS activity and stimulation of mitochondrial β-oxidation via CPT1 [53,54,55,56,57,58]. At the intestinal level, the combined action of soluble fiber, mucilage, and phenolic compounds may contribute to reduced lipid absorption through inhibition of NPC1L1 and sequestration of dietary lipids, thereby promoting cholesterol catabolism into bile acids via CYP7A1 [59,60,61]. The increase in HDL-C may also be related to activation of the reverse cholesterol transport pathway through PPARα signaling and enhanced expression of ApoA-I together with ABCA1/ABCG1 cholesterol transporters [62,63,64]. Importantly, these mechanisms were not directly investigated in the current study. Therefore, they should be considered as plausible mechanisms based on previous evidence rather than demonstrated mechanisms of action of OCE.

Simvastatin was chosen as the reference positive control due to its well-known pharmacological activity as an HMGCR inhibitor and its frequent use as a comparator in experimental models of diet-induced hyperlipidemia [65]. Its mode of action directly targets endogenous cholesterol biosynthesis [66], making it suitable for benchmarking the lipid-lowering effect of OCE. Among statins, differences in lipophilicity, membrane interaction, intestinal absorption and tissue distribution may influence their biological profiles; simvastatin is relatively lipophilic, whereas pravastatin and rosuvastatin are more hydrophilic, which may contribute to differences in their tissue distribution and pharmacological profiles [67]. Notably, the study was not designed to compare the efficacy or metabolic safety profiles of different statins. The observed improvement in fasting glucose, HDL-C, and hepatic MDA with OCE suggests that the biological effects of OCE may extend beyond cholesterol lowering alone. However, it remains to be determined whether these additional effects are specific to OCE, and whether they differ from those of other statins, which would require direct comparative studies.

In addition to its lipid-lowering activity, OCE may have beneficial cardiovascular implications. Hyperlipidemia, hyperglycemia, hepatic steatosis and oxidative stress are risk factors associated with CVD [68,69,70]. In the present study, OCE improved the lipid profile, restored fasting glucose levels, attenuated hepatic steatosis and reduced oxidative stress, consistent with an overall improvement in several cardiometabolic parameters. However, since vascular pathology and cardiac function were not directly measured, these findings should be interpreted as indicating a possible reduction in cardiovascular risk and not necessarily as a cardioprotective effect. More research is needed to confirm these potential heart benefits. Histological examination further corroborated these findings, as OCE treatment was associated with reduced hepatic lipid accumulation and improved overall liver morphology compared to the HFD group. However, the histological assessment was based on representative microscopic observations of H&E-stained sections, instead of a standardized quantitative or semi-quantitative scoring [71]. Therefore, these results should be regarded as supportive morphological evidence and not as conclusive quantitative evidence of histological improvement.

Besides phenolic compounds, untargeted metabolomics revealed a high relative abundance of primary metabolites, especially organic acids, such as citric, galactaric, gluconic, aconitic, malonic, and malic acids, in OCE. Their relatively high LC-MS signal intensities should not be considered as direct evidence that they are the principal bioactive constituents, but these metabolites may contribute to the overall biological activity of OCE via complementary metabolic mechanisms. Citric and malic acids are key intermediates of the tricarboxylic acid (TCA) cycle and related shuttle systems and thus contribute to mitochondrial energy metabolism, cellular redox homeostasis and limiting excessive ROS production under metabolic stress [72,73]. In addition, it has been reported that citric acid attenuates hepatic lipid peroxidation by decreasing MDA accumulation and increasing the endogenous antioxidant defenses, including SOD and GSH-Px activities [74,75]. However, the beneficial effects observed in the present study may reflect the combined contribution of primary metabolites such as organic acids and amino acids, together with specialized phytochemicals, especially phenolic acids and flavonoids, rather than the action of any single metabolite.

The antioxidant assays further support the biological activity of OCE. The crude extract contained higher concentrations of total phenolics, flavonoids, and condensed tannins than the corresponding fractions, indicating that aqueous extraction efficiently recovered phenolic and other antioxidant phytochemicals [76]. Following liquid–liquid partitioning, phenolic acids were mainly retained in the aqueous fraction, whereas moderately polar flavonoids were enriched in the ethyl acetate fraction, consistent with their polarity and previously reported partitioning behavior [77]. These bioactive compounds likely contribute to radical-scavenging activity and may limit lipid peroxidation, thereby potentially contributing to the protection of hepatic and vascular tissues from HFD-induced oxidative stress [78,79]. In agreement with previous mechanistic studies, this antioxidant effect may involve activation of the Keap1/Nrf2 signaling pathway, leading to increased expression of endogenous antioxidant enzymes such as SOD, CAT, GPx, and HO-1 while suppressing NF-κB-mediated inflammatory signaling [12,80,81]. However, these signaling pathways were not directly studied in the present study. This interpretation is further supported by the marked reduction in hepatic MDA levels observed following OCE administration.

Molecular docking analysis provided additional structural evidence consistent with the experimental findings. Several flavonoids identified in OCE displayed favorable predicted binding affinities toward proteins involved in cholesterol homeostasis and lipid metabolism. In particular, isorhamnetin-3-*O*-rutinoside showed strong predicted interactions with HMG-CoA reductase, whereas quercetin-3-*O*-rutinoside exhibited favorable binding to FAS and FXR. Kaempferol showed high predicted affinity for PPARα and NPC1L1, while naringenin interacted favorably with LXRα. Together, these results provide plausible molecular hypotheses that may help explain the lipid-lowering effects observed in vivo [46,52,60,64,82]. Nevertheless, molecular docking remains a predictive computational approach and cannot be considered direct evidence of enzyme inhibition or receptor modulation under physiological conditions, as in silico models cannot fully replicate complex in vivo pharmacokinetic dynamics, tissue distribution, and systemic physiological interactions [83,84].

The ADMET analysis provided preliminary computational information regarding the predicted absorption and toxicological characteristics of selected putatively annotated metabolites. Most aglycone flavonoids complied with Lipinski’s rule of five and exhibited favorable predicted oral absorption. Conversely, glycosylated derivatives showed lower intestinal permeability, which is consistent with their physicochemical properties and their potential conversion into absorbable aglycones by the intestinal microbiota [85,86]. Predicted toxicological profiles indicated generally low predicted acute toxicity for most metabolites, with several compounds exhibiting predicted LD_50_ values up to 5000 mg/kg. In addition, most phytochemicals were predicted to be non-mutagenic, non-hepatotoxic, and inactive toward CYP3A4 and CYP2D6 inhibition, suggesting a potentially limited interaction liability for several of the annotated metabolites [87].

Some limitations of the present study should be noted. Untargeted UHPLC–MS/MS resulted in broad metabolite coverage, although the annotated compounds were assigned MSI Level 2 confidence without absolute quantification with authentic reference standards under the same conditions. Thus, peak abundances reflect relative signal intensities and not absolute metabolite concentrations. Furthermore, the mechanisms proposed by computational docking and ADMET analyses are still predictive and need to be validated using targeted enzymatic assays and hepatic gene/protein expression analyses such as RT-qPCR and Western blotting. Inflammatory mediators and NF-κB signaling were not directly assessed, and histological assessment was based on H&E-stained sections without standardized quantitative scoring. Furthermore, the use of a single OCE dose (200 mg/kg b.w.) prevented assessment of a dose–response relationship, and potential cardiovascular benefits were inferred from cardiometabolic parameters rather than assessed directly. Finally, although no overt signs of toxicity were noted during the 9-week OCE treatment period under the experimental conditions used, longer-term toxicological and pharmacokinetic studies are required. Thus, future studies should incorporate targeted metabolomics, dose–response experiments, molecular and inflammatory profiling, standardized histopathology, and longer-term safety and pharmacokinetic assessments to further define the mechanisms and translational potential of OCE.

## 5. Conclusions

The present study showed that the aqueous extract of *O. ficus-indica* cladodes is a promising natural candidate for the management of hyperlipidemia and associated hepatic lipid accumulation. In the present work, we integrated in vitro antioxidant assays, in vivo pharmacological evaluation, untargeted metabolomic profiling and in silico molecular docking to provide complementary evidence for the biological potential of this underexploited plant resource.

From an industrial point of view, the present results highlight the potential of *O. ficus-indica* cladodes as a sustainable, climate-resilient agricultural resource for the development of standardized herbal preparations, nutraceuticals and functional foods. The use of an aqueous extraction process may also support its potential application in food and botanical-product industries.

Future studies should focus on experimental validation of the proposed molecular mechanisms through gene and protein expression studies (e.g., RT-qPCR and Western blotting); investigation of synergistic interactions among the major phytochemicals and the role of gut microbiota in their biotransformation; optimization of formulation strategies to improve bioavailability; and well-designed clinical studies to establish long-term safety, efficacy, and translational relevance. These studies will help to determine whether OCE can be further developed as an evidence-based nutritional or nutraceutical approach for the management of hyperlipidemia and associated cardiometabolic disorders.

## Figures and Tables

**Table 1:** Distribution of total phenolics, flavonoids, and condensed tannins in the crude extract and the fractions obtained by liquid–liquid partitioning.

Fraction	Total Phenolic Content (mg GAE/g DM)	Total Flavonoid Content (mg RE/g DM)	Tannins (mg CE/g DM)
Crude extract (OCE)	316.56 ± 0.65 ^c^	33.36 ± 1.03 ^c^	47.67 ± 2.84 ^b^
Aqueous fraction	159.01 ± 2.54 ^b^	10.49 ± 1.11 ^a^	21.00 ± 1.50 ^a^
Ethyl acetate fraction	120.81 ± 1.12 ^a^	16.28 ± 0.70 ^b^	19.50 ± 1.80 ^a^

The findings are shown as mean ± SD. One-way ANOVA and Tukey’s post hoc test were used. Values with different superscript letters (a–c) within the same column are significantly different (*p* < 0.05). Values with at least one common letter are not significantly different. GAE: Gallic acid equivalent; RE: Rutin equivalent; CE: Catechin equivalent; DM: Dry matter.

**Table 2:** Tentatively identified metabolites in *O. ficus-indica* cladode extract by UHPLC–Orbitrap IQ-X Tribrid MS/MS operating in negative ion mode ([M − H]^−^).

No.	Metabolites	Molecular Formula
Phenolic acids and derivatives		
1	Piscidic acid	C_11_H_12_O_7_
2	4,6-dihydroxy-3-(1-hydroxyethyl)-5-methoxy-3H-2-benzofuran-1-one	C_11_H_12_O_6_
3	3-(3-Hydroxyphenyl)propionic acid	C_9_H_10_O_3_
4	4-Hydroxybenzoic acid	C_7_H_6_O_3_
5	Caffeic acid	C_9_H_8_O_4_
6	3-Feruloylquinic acid	C_17_H_20_O_9_
7	*p*-Coumaric acid	C_9_H_8_O_3_
8	*p*-Coumaric acid glucoside	C_15_H_18_O_8_
9	3,4-Dihydroxyallylbenzene 3,4-di-*O*-glucoside	C_21_H_30_O_12_
10	2-*O*-Glucosyloxy-4-methoxycinnamic acid	C_16_H_20_O_9_
11	Osmanthuside H	C_19_H_28_O_11_
12	Pyrocatechol	C_6_H_6_O_2_
13	Feruloyl quinic acid	C_17_H_20_O_9_
14	Salicylic acid β-glucoside	C_13_H_16_O_8_
15	1-*O*-Feruloylglucose	C_16_H_20_O_9_
16	*p*-Coumaryl alcohol	C_9_H_10_O_2_
17	Salicylic acid	C_7_H_6_O_3_
18	Vanillic acid + *O*-sulfonateHex	C_14_H_18_O_12_S
19	(E/Z)-ferulic acid	C_10_H_10_O_4_
20	Caffeic acid 3-β-D-glucuronide	C_15_H_16_O_10_
21	Gallic acid	C_7_H_6_O_5_
22	Cinnamic acid	C_9_H_8_O_2_
Flavonoids and derivatives		
23	Isorhamnetin	C_16_H_12_O_7_
24	Isorhamnetin-3-*O*-acetylglucoside	C_24_H_24_O_13_
25	Naringenin	C_15_H_12_O_5_
26	Quercimeritrin trihydrate	C_21_H_26_O_15_
27	Quercetin-3-*O*-α-L rhamnopyranosyl(1–2)-β D-glucopyranoside-7-*O*-α-L-rhamnopyranoside	C_33_H_40_O_20_
28	Taxifolin-3-glucoside	C_21_H_22_O_12_
29	Quercetin-3-*O*-rutinoside	C_27_H_30_O_16_
30	Xanthorhamnin	C_34_H_42_O_20_
31	Quercetin-3′-glucoside	C_21_H_20_O_12_
32	Licoagroside B	C_18_H_24_O_12_
33	Isorhamnetin-3-*O*-rutinoside	C_28_H_32_O_16_
34	2′,3,5,6′,7-Pentahydroxyflavanone	C_15_H_12_O_7_
35	3″-Galloylquercitrin	C_28_H_24_O_15_
36	Genistein 7-glucoside	C_21_H_20_O_10_
37	5-*O*-Methyllicoricidin	C_27_H_34_O_5_
38	Wedelolactone	C_16_H_10_O_7_
39	Dihydrokaempferol	C_15_H_12_O_6_
40	Acacetin	C_16_H_12_O_5_
41	Flavone base + 4*O*, *O*-MalonylHex	C_24_H_22_O_14_
42	Quercetin 3-(2R-apiosylrutinoside)	C_32_H_38_O_20_
43	Isorhamnetin 3-galactoside	C_22_H_22_O_12_
44	Kaempferol 7-*O*-glucoside	C_21_H_20_O_11_
45	Formononetin 7-*O*-β-D-glucopyranoside	C_22_H_22_O_9_
46	5,7-Dihydroxyflavone	C_15_H_10_O_4_
47	Kaempferol 3-*O*-vicianoside	C_26_H_28_O_15_
48	Isobavachin	C_20_H_20_O_4_
49	Kaempferol	C_15_H_10_O_6_
50	Robinin	C_33_H_40_O_19_
51	Quercetin	C_15_H_10_O_7_
52	4′-Hydroxyflavone	C_15_H_10_O_3_
Organic acid, lipids and amino acid		
53	Citric acid	C_6_H_8_O_7_
54	Galactaric acid	C_6_H_10_O_8_
55	Gluconic acid	C_6_H_12_O_7_
56	2-Furoic acid	C_5_H_4_O_3_
57	D-Glucuronic acid	C_6_H_10_O_7_
58	Malonic acid	C_3_H_4_O_4_
59	Pinellic acid	C_18_H_34_O_5_
60	(E)-Aconitic acid	C_6_H_6_O_6_
61	Crotonic acid	C_4_H_6_O_2_
62	PG *O*-11:0_24:4	C_41_H_75_O_9_P
63	L-threonic acid	C_4_H_8_O_5_
64	2-Isopropylorganic acid	C_7_H_12_O_5_
65	Tetrathermic acid	C_18_H_32_O_5_
66	L-citramalic acid	C_5_H_8_O_5_
67	3-Hydroxypropanoic acid	C_3_H_6_O_3_
68	Quinic acid	C_7_H_12_O_6_
69	Tiglic acid	C_5_H_8_O_2_
70	3-Hydroxy-3-methylglutaric acid	C_6_H_10_O_5_
71	(15Z)-9,12,13-Trihydroxy-15-octadecenoic acid	C_18_H_34_O_5_
72	N-Acetyltryptophan	C_13_H_14_N_2_O_3_
73	D-Glutamic acid	C_5_H_9_NO_4_
74	Azelaic acid	C_9_H_16_O_4_
75	L-Pyroglutamic acid	C_5_H_7_NO_3_
76	(±)-2-Hydroxyisovaleric acid	C_5_H_10_O_3_
77	Glutaric acid	C_5_H_8_O_4_
78	N-Isovalerylglycine	C_7_H_13_NO_3_
79	LPE 2:0	C_7_H_16_NO_7_P
80	D-pantothenic acid	C_9_H_17_NO_5_
81	Ascorbic acid	C_6_H_8_O_6_
82	Malic acid	C_4_H_6_O_5_
83	DL-Tryptophan	C_11_H_12_N_2_O_2_
84	L-histidine	C_6_H_9_N_3_O_2_
85	Lysophosphatidic acid	C_21_H_41_O_7_P
86	D-alanine	C_3_H_7_NO_2_
Other metabolites		
87	Inosine	C_10_H_12_N_4_O_5_
88	D-Galactose	C_6_H_12_O_6_
89	Galactitol	C_6_H_14_O_6_
90	D-mannitol	C_6_H_14_O_6_
91	Lyxose	C_5_H_10_O_5_
92	3-Oxo-α-ionol 9-glucoside	C_19_H_30_O_7_
93	Stachyose	C_24_H_42_O_21_
94	δ-Gluconolactone	C_6_H_10_O_6_
95	Maltitol	C_12_H_24_O_11_
96	Lumazine	C_6_H_4_N_4_O_2_
97	Sibiricose A1	C_23_H_32_O_15_
98	Isomaltulose	C_12_H_22_O_11_
99	D-Arabitol	C_5_H_12_O_5_
100	D-Mannose	C_6_H_12_O_6_
101	Raffinose	C_18_H_32_O_16_
102	(+)-syringaresinol β-D-glucoside	C_28_H_36_O_13_

**Table 3:** Body weight gain and organ weights (liver, kidneys, and heart) of mice treated with OCE or simvastatin during high-fat diet feeding.

Group	ΔBWG (g)	Liver (g)	Kidneys (g)	Heart (g)
NLC	1.92 ± 0.38 ^a^	1.58 ± 0.03 ^a^	0.44 ± 0.04 ^ab^	0.17 ± 0.02 ^a^
HLC	5.88 ± 0.94 ^c^	1.99 ± 0.25 ^b^	0.42 ± 0.02 ^a^	0.16 ± 0.01 ^a^
OCEG	3.92 ± 0.17 ^b^	1.85 ± 0.10 ^ab^	0.43 ± 0.02 ^a^	0.14 ± 0.01 ^a^
SVTG	3.80 ± 0.29 ^b^	1.87 ± 0.27 ^ab^	0.50 ± 0.03 ^b^	0.15 ± 0.01 ^a^

Values are expressed as mean ± SD (*n* = 8). Different superscript letters within the same column indicate statistically significant differences (*p* < 0.05; one-way ANOVA followed by Tukey’s post hoc test). NLC, normal control; HLC, hyperlipidemic control; OCEG, *Opuntia* cladode extract-treated group; SVTG, simvastatin-treated group; ΔBWG, body weight gain.

## Data Availability

Data are contained within the article. Further inquiries can be directed to the corresponding authors.

## Supplementary Materials

The following supporting information can be downloaded at: https://www.mdpi.com/article/10.3390/nu18172766/s1, Table S1. Putative identification of metabolites in O. ficus-indica cladode extract analyzed by UHPLC–Orbitrap IQ-X Tribrid MS/MS in negative ion mode ([M − H]−); Table S2. Predicted physicochemical and pharmacokinetic properties of the major Opuntia ficus-indica bioactive compounds and simvastatin; Table S3. Predicted toxicological profile of the major O. ficus-indica bioactive compounds and simvastatin.

## Abbreviations

The following abbreviations are used in this manuscript:

ABCA1	ATP-binding cassette transporter A
ACAT	Acyl-CoA:cholesterol acyltransferase
ACLY	ATP citrate lyase
ADMET	Absorption, Distribution, Metabolism, Excretion, and Toxicity
AMPK	AMP-activated protein kinase
ApoB	Apolipoprotein B
CAT	Catalase
CETP	Cholesteryl ester transfer protein
CYS	Cysteine
DPPH	2,2-diphenyl-1-picrylhydrazyl
EC50	Effective concentration
FXR	Farnesoid X receptor
GLN	Glutamine
GLU	Glutamic acid
GPx	Glutathione peroxidase
HO-1	Heme oxygenase-1
IRS-1/PI3K	Insulin receptor substrate 1/Phosphoinositide 3-kinase
LYS	Lysine
mTOR	Mammalian target of rapamycin
Nrf2	Nuclear factor erythroid 2-related factor 2
OECD	Organization for Economic Co-operation and Development
PCSK9	Proprotein convertase subtilisin/kexin type 9
PDBQT	Protein Data Bank, Partial Charge (Q), and Atom Type (T)
SOD	Superoxide dismutase
SREBP-1	Sterol regulatory element-binding protein 1
TPSA	Topological Polar Surface Area
UHPLC	Ultra-High-Performance Liquid Chromatography
VLDL	Very-low-density lipoprotein
Wnt	Wingless-related integration site
