# Supplementary material for: Polyphenol-Rich Opuntia ficus-indica Cladodes: An Integrated Metabolomic, In Vivo and In Silico Study Supporting Their Hypolipidemic and Hepatoprotective Effects
Source: Nutrients. 2026 Aug 24;18(17):2766. doi: 10.3390/nu18172766 (PMC13567411; doi:10.3390/nu18172766)
Supplement: Supplementary file 1 [file nutrients-18-02766-s001.zip › nutrients-4458414-supplementary.pdf]

Table S1. Putative identification of metabolites in *O. ficus-indica* cladode extract analyzed by UHPLC–Orbitrap IQ-X Tribrid MS/MS in negative ion mode ([M − H]−).

**No.**	**Metabolites**	**RT**	**[M – H]⁻ (m/z)**	**Molecular formula**	**Peak Area**	**Major MS/MS fragments (m/z)**
			**Phenolic acids and derivatives**			
**1**	Piscidic acid	3.87	255.0510	$C_{11}H_{12}O_7$	1.32×10^9	163.040, 135.045, 133.029
**2**	4,6-dihydroxy-3-(1-hydroxyethyl)-5-methoxy-3H-2-benzofuran-1-one	4.81	239.0561	$C_{11}H_{12}O_6$	5.42×10^7	177.056, 133.066, 87.009
**3**	3-(3-Hydroxyphenyl)propionic acid	3,86	165.0557	$C_9H_{10}O_3$	3.11×10^7	121.041, 119.050, 58.006
**4**	4-Hydroxybenzoic acid	3.07	137.0244	$C_7H_6O_3$	4.82×10^6	93.035, 136.892, 51.060
**5**	Caffeic acid	3.86	179.0349	$C_9H_8O_4$	4.79×10^6	135.045, 87.009, 134.037
**6**	3-Feruloylquinic acid	1.88	367.0519	$C_{17}H_{20}O_9$	4.69×10^6	163.037, 324.331, 145.028
**7**	p-Coumaric acid	4.40	163.0401	$C_9H_8O_3$	3.85×10^6	119.050, 117.034, 91.055
**8**	p-Coumaric acid glucoside	4.39	325.0931	$C_{15}H_{18}O_8$	3.76×10^6	163.040, 119.050, 147.045
**9**	3,4-Dihydroxyallylbenzene 3,4-di-*O*-glucoside	1.44	473.1509	$C_{21}H_{30}O_{12}$	3.02×10^6	85.029, 205.072, 397.135
**10**	2-*O*-Glucosyloxy-4-methoxycinnamic acid	3.68	356.1107	$C_{16}H_{20}O_9$	2.66×10^6	177.056, 341.088, 101.024
**11**	Osmanthuside H	3.62	431.1559	$C_{19}H_{28}O_{11}$	1.88×10^6	161.045, 59.014, 281.067
**12**	Pyrocatechol	3.33	109.0295	$C_6H_6O_2$	1.63×10^6	108.021, 81.034, 91.019
**13**	Feruloyl quinic acid	1.21	367.1059	$C_{17}H_{20}O_9$	1.62×10^6	157.031, 71.014, 85.029
**14**	Salicylic acid β-glucoside	3.09	299.0777	$C_{13}H_{16}O_8$	1.35×10^6	71.014, 121.029, 83.014
**15**	1-*O*-Feruloylglucose	4.69	355.1037	$C_{16}H_{20}O_9$	1.06×10^6	149.061, 159.045, 191.034
**16**	p-Coumaryl alcohol	3.88	149.0608	$C_9H_{10}O_2$	8.41×10^5	93.034, 119.050, 75.009
**17**	Salicylic acid	8.33	137.0244	$C_7H_6O_3$	6.38×10^5	81.837, 93.034, 101.923
**18**	Vanillic acid + *O*-sulfonateHex	6.36	409.0448	$C_{14}H_{18}O_{12}S$	6.34×10^5	223.0, 164.986, 209.045
**19**	(E/Z)-ferulic acid	4.26	193.0507	$C_{10}H_{10}O_4$	6.10×10^5	134.037, 178.027, 102.933
**20**	Caffeic acid 3-β-D-glucuronide	5.11	355.0674	$C_{15}H_{16}O_{10}$	5.49×10^5	191.020, 147.030, 163.040
**21**	Gallic acid	8.24	169.0143	$C_7H_6O_5$	3.40×10^5	125.024, 107.014, 57.034
**22**	Cinnamic acid	8,01	147,0451	$C_9H_8O_2$	$1{,}47\times10^5$	147.045, 102.93, 100.93

			**Flavonoids and derivatives**			
**23**	Isorhamnetin	9.74	315.0512	$C_{16}H_{12}O_7$	1.20×10^{7}	300.028, 271.024, 151.004
**24**	Isorhamnetin-3-*O*-acetylglucoside	3.85	519.0951	$C_{24}H_{24}O_{13}$	8.54×10^{6}	429.100, 147.045, 501.084
**25**	Naringenin	3.36	271.0460	$C_{15}H_{12}O_5$	4.87×10^{6}	151.004, 93.035, 83.014
**26**	Quercimeritrin trihydrate	1.29	517.1409	$C_{21}H_{26}O_{15}$	4.81×10^{6}	71.014, 113.024, 293.088
**27**	Quercetin-3-*O*-α-L rhamnopyranosyl(1-2)- β D-glucopyranoside-7-*O*- α -L-rhamnopyranoside	5.80	755.2045	$C_{33}H_{40}O_{20}$	4.64×10^{6}	313.035, 285.040, 295.061
**28**	Taxifolin-3-glucoside	3.86	466.1110	$C_{21}H_{22}O_{12}$	4.61×10^{6}	165.056, 149.061, 211.061
**29**	Quercetin-3-*O*-rutinoside	5.98	609.1462	$C_{27}H_{30}O_{16}$	4.42×10^{6}	301.036, 151.004, 271.025
**30**	Xanthorhamnin	5.73	769.2203	$C_{34}H_{42}O_{20}$	3.37×10^{6}	314.044, 287.056, 356.054
**31**	Quercetin-3'-glucoside	3.87	509.0937	$C_{21}H_{20}O_{12}$	3.18×10^{6}	403.104, 313.108, 343.083
**32**	Licoagroside B	11.7	431.1845	$C_{18}H_{24}O_{12}$	3.15×10^{6}	152.996, 78.959, 150.980
**33**	Isorhamnetin-3-O-rutinoside	6.49	623.1611	$C_{28}H_{32}O_{16}$	3.03×10^{6}	315.051, 287.056, 329.067
**34**	2',3,5,6',7-Pentahydroxyflavanone	6.57	303.0514	$C_{15}H_{12}O_7$	2.35×10^{6}	285.040, 175.040, 217.051
**35**	3''-Galloylquercitrin	3.87	600.1112	$C_{28}H_{24}O_{15}$	1.89×10^{6}	309.947, 308.930, 294.951
**36**	Genistein 7-glucoside	9.07	431.0986	$C_{21}H_{20}O_{10}$	1.81×10^{6}	134.037, 178.027, 147.045
**37**	5-*O*-Methyllicoricidin	4.52	437.2394	$C_{27}H_{34}O_5$	1.71×10^{6}	113.024, 244.976, 99.009
**38**	Wedelolactone	9.74	313.0358	$C_{16}H_{10}O_7$	1.70×10^{6}	298.012, 226.027, 251.202
**39**	Dihydrokaempferol	7.49	287.0562	$C_{15}H_{12}O_6$	1.56×10^{6}	259.061, 151.004, 201.056
**40**	Acacetin	1.23	283.1038	$C_{16}H_{12}O_5$	1.31×10^{6}	101.024, 71.014, 89.024
**41**	Flavone base + 4*O*, *O*-MalonylHex	3.87	533.0912	$C_{24}H_{22}O_{14}$	1.25×10^{6}	193.051, 443.251, 149.395
**42**	Quercetin 3-(2R-apiosylrutinoside)	5.40	741.1894	$C_{32}H_{38}O_{20}$	1.16×10^{6}	300.028, 151.004, 273.041
**43**	Isorhamnetin 3-galactoside	6.79	477.1039	$C_{22}H_{22}O_{12}$	1.14×10^{6}	314.044, 204.043, 449.109
**44**	Kaempferol 7-*O*-glucoside	4.24	447.1148	$C_{21}H_{20}O_{11}$	1.02×10^{6}	152.011, 401.181, 101.024
**45**	Formononetin 7-*O*-β-D-glucopyranoside	1.38	429.12515	$C_{22}H_{22}O_9$	9.55×10^{5}	71.014, 97.030, 89.024
**46**	5,7-Dihydroxyflavone	1.28	253.0567	$C_{15}H_{10}O_4$	8.81×10^{5}	71.014, 97.030, 89.024
**47**	Kaempferol 3-*O*-vicianoside	6.17	579.1359	$C_{26}H_{28}O_{15}$	8.23×10^{5}	284.033, 237.077, 279.088
**48**	Isobavachin	3.68	323.1349	$C_{20}H_{20}O_4$	7.74×10^{5}	59.014, 95.014, 85.029

**49**	Kaempferol	9.65	285.0405	$C_{15}H_{10}O_6$	7.54×10^5	151.004, 257.045, 107.014
**50**	Robinin	5.67	739.2085	$C_{33}H_{40}O_{19}$	7.50×10^5	283.025, 257.046, 151.004
**51**	Quercetin	8.56	301.0355	$C_{15}H_{10}O_7$	5.59×10^5	151.004, 178.999, 273.041
**52**	4'-Hydroxyflavone	1.76	237.0617	$C_{15}H_{10}O_3$	5.09×10^5	75.009, 71.014, 57.034
			**Organic acid, lipids and amino acid**			
**53**	Citric acid	2.08	191.0197	$C_6H_8O_7$	3.72×10^8	111.009, 87.009, 102.949
**54**	Galactaric acid	1.32	209.0302	$C_6H_{10}O_8$	1.64×10^8	85.030, 57.035, 129.019
**55**	Gluconic acid	1.27	195.0511	$C_6H_{12}O_7$	1.01×10^8	75.009, 129.019, 99.009
**56**	2-Furoic acid	1.91	111.0088	$C_5H_4O_3$	8.02×10^7	111.009, 83.014, 62.214
**57**	D-Glucuronic acid	1. 64	193.0356	$C_6H_{10}O_7$	2.91×10^7	59.014, 89.024, 101.024
**58**	Malonic acid	1.65	103.0037	$C_3H_4O_4$	1.55×10^7	59.014, 103.004
**59**	Pinellic acid	9.46	329.2334	$C_{18}H_{34}O_5$	1.33×10^7	329.234, 171.103, 157.123
**60**	(E)-Aconitic acid	1.91	173.0092	$C_6H_6O_6$	1.07×10^7	55.400, 73.691, 85.029
**61**	Crotonic acid	1.31	85.0295	$C_4H_6O_2$	8.71×10^6	46.259, 55.018
**62**	PG *O*-11:0_24:4	15.3	741.4992	$C_{41}H_{75}O_9P$	6.18×10^6	85.953, 237.906, 471.206
**63**	L-threonic acid	1.33	135.0299	$C_4H_8O_5$	5.38×10^6	89.024, 55.019, 134.895
**64**	2-Isopropylmalic acid	4.46	175.0612	$C_7H_{12}O_5$	4.91×10^6	115.040, 131.071, 157.051
**65**	Tetrathermic acid	8.95	327.2178	$C_{18}H_{32}O_5$	3.22×10^6	171.103, 85.029, 309.207
**66**	L-citramalic acid	2.17	147.0299	$C_5H_8O_5$	2.35×10^6	87.009, 129.019, 43.019
**67**	3-Hydroxypropanoic acid	5.19	89.0244	$C_3H_6O_3$	2.21×10^6	49.393, 89.024
**68**	Quinic acid	1.25	383.1199	$C_7H_{12}O_6$	1.66×10^6	159.030, 101.024, 129.019
**69**	Tiglic acid	2.13	99.0087	$C_5H_8O_2$	1.37×10^6	98.956, 55.019, 78.959
**70**	3-Hydroxy-3-methylglutaric acid	2.46	161.0457	$C_6H_{10}O_5$	1.28×10^6	99.045, 160.842, 97.029
**71**	(15Z)-9,12,13-Trihydroxy-15-octadecenoic acid	10.6	329.2338	$C_{18}H_{34}O_5$	1.21×10^6	201.113, 311.223, 275.202
**72**	N-Acetyltryptophan	6.68	245.0933	$C_{13}H_{14}N_2O_3$	1.07×10^6	203.083, 74.025, 116.035
**73**	D-Glutamic acid	1.13	146.0459	$C_5H_9NO_4$	1.06×10^6	102.056, 128.035, 146.046
**74**	Azelaic acid	6.99	187.0977	$C_9H_{16}O_4$	1.03×10^6	125.097, 83.050, 116.051
**75**	L-Pyroglutamic acid	1.86	128.0354	$C_5H_7NO_3$	1.02×10^6	80.986, 128.035
**76**	(±)-2-Hydroxyisovaleric acid	3.65	117.0557	$C_5H_{10}O_3$	9.36×10^5	59.013, 71.050, 115.039

**77**	Glutaric acid	2.91	131.0350	$C_5H_8O_4$	7.44×10^5	59.013, 87.044, 113.024
**78**	N-Isovalerylglycine	4.01	158.0822	$C_7H_{13}NO_3$	7.41×10^5	89.693, 153.594
**79**	LPE 2:0	3.98	256.0544	$C_7H_{16}NO_7P$	6.57×10^5	124.048, 194.054, 165.056
**80**	D-pantothenic acid	3.18	218.1034	$C_9H_{17}NO_5$	6.48×10^5	88.040, 71.014, 116.035
**81**	Ascorbic acid	1.71	175.0248	$C_6H_8O_6$	6.42×10^5	87.009, 57.034, 83.014
**82**	Malic acid	1.49	133.0141	$C_4H_6O_5$	6.38×10^5	59.013, 71.013, 109.759
**83**	DL-Tryptophan	9.46	204.0898	$C_{11}H_{12}N_2O_2$	5.61×10^5	100.933, 293.049, 357.117
**84**	L-histidine	1.06	154.0622	$C_6H_9N_3O_2$	2.94×10^5	93.046, 154.062, 110.072
**85**	Lysophosphatidic acid	15.6	435.2518	$C_{21}H_{41}O_7P$	2.71×10^5	117.509, 171.006, 200.949
**86**	D-alanine	1.14	88.0403	$C_3H_7NO_2$	2.03×10^5	88.040
			**Other metabolites**			
**87**	Inosine	1.39	267.0723	$C_{10}H_{12}N_4O_5$	4.90×10^6	113.024, 71.014, 9.014
**88**	D-Galactose	1.62	179.0561	$C_6H_{12}O_6$	7.96×10^6	59.014, 89.024, 83.014
**89**	Galactitol	1.18	81.0717	$C_6H_{14}O_6$	3.70×10^6	71.014, 59.014, 83.014
**90**	D-mannitol	1.38	181.0717	$C_6H_{14}O_6$	3.68×10^6	71.014, 89.024, 71.014
**91**	Lyxose	1.42	149.0455	$C_5H_{10}O_5$	3.31×10^6	131.035163.014, 73.029
**92**	3-Oxo-α-ionol 9-glucoside	6.18	370.1991	$C_{19}H_{30}O_7$	2.76×10^6	59.014, 85.029, 137.097
**93**	Stachyose	1.33	665.2149	$C_{24}H_{42}O_{21}$	2.43×10^6	101.024, 97.029, 89.024
**94**	δ-Gluconolactone	1.26	177.0406	$C_6H_{10}O_6$	2.22×10^6	99.009, 71.014, 57.034
**95**	Maltitol	1.20	343.1247	$C_{12}H_{24}O_{11}$	2.11×10^6	101.024, 89.024, 59.014
**96**	Lumazine	1.42	163.0248	$C_6H_4N_4O_2$	2.11×10^6	101.024, 72.993, 71.014
**97**	Sibiricose A1	5.81	547.1710	$C_{23}H_{32}O_{15}$	2.05×10^6	79.957, 89.024, 75.009
**98**	Isomaltulose	1.41	341.1090	$C_{12}H_{22}O_{11}$	1.94×10^6	89.024, 101.024, 71.014
**99**	D-Arabitol	1.21	151.0612	$C_5H_{12}O_5$	1.91×10^6	71.014, 101.024, 73.029
**100**	D-Mannose	1.19	180.0631	$C_6H_{12}O_6$	1.75×10^6	114.056, 131.046, 171.078
**101**	Raffinose	1.22	504.1690	$C_{18}H_{32}O_{16}$	1.31×10^6	97.029, 71.014, 425.130
**102**	(+)-syringaresinol β-D-glucoside	6.36	579.2088	$C_{28}H_{36}O_{13}$	1.23×10^6	96.960, 193.051, 356.126

Compounds are prioritized based on peak areas. RT: retention time (min); MS/MS fragments: product ions utilized for annotation.

Table S2. Predicted physicochemical and pharmacokinetic properties of the major *Opuntia ficus-indica* bioactive compounds and simvastatin.

CID	Compound	LogP	TPSA (Å²)	Lipinski Rule	HIA	BBB Penetration	CYP3A4 Inhibition	Drug Clearance
**10038020**	Piscidic acid	−0.80	135.29	4/4	0.87	0.33	Inactive	13.22
**689043**	Caffeic acid	1.20	77.76	4/4	0.98	0.39	Inactive	18.67
**637542**	*p*-Coumaric acid	1.49	57.53	4/4	0.99	0.41	Inactive	28.20
**5481663**	Isorhamnetin-3-*O*-rutinoside	−1.38	258.43	1/4	0.11	0.06	Inactive	26.18
**5280805**	Quercetin-3-*O*-rutinoside	−1.69	269.43	1/4	0.09	0.06	Inactive	25.57
**122850**	Dihydrokaempferol	1.48	107.22	4/4	1.00	0.09	Inactive	72.68
**5281654**	Isorhamnetin	2.29	120.36	4/4	1.00	0.13	Inactive	86.09
**5280343**	Quercetin	1.99	131.36	4/4	1.00	0.05	Inactive	0.08
**439246**	Naringenin	2.51	86.99	4/4	1.00	0.23	Active	13.94
**5280863**	Kaempferol	2.28	111.13	4/4	1.00	0.07	Inactive	6.22
**54454**	Simvastatin	4.59	72.83	4/4	1.00	0.52	Active	48.84

ADMET properties were predicted using ADMET-AI (v1.3.1). HIA, human intestinal absorption; BBB, blood–brain barrier; TPSA, topological polar surface area; CYP3A4, cytochrome P450 3A4.

Table S3. Predicted toxicological profile of the major *O. ficus-indica* bioactive compounds and simvastatin.

CID	Compound	LD_{50} (mg/kg)	Hepatotoxicity	Nephrotoxicity	Mutagenicity
**10038020**	Piscidic acid	1320	Inactive	Inactive	Inactive
**689043**	Caffeic acid	2980	Inactive	Active	Inactive
**637542**	*p*-Coumaric acid	2850	Inactive	Active	Inactive
**5481663**	Isorhamnetin-3-*O*-rutinoside	5000	Inactive	Active	Inactive
**5280805**	Quercetin-3-*O*-rutinoside	5000	Inactive	Active	Inactive
**122850**	Dihydrokaempferol	2000	Inactive	Active	Inactive
**5281654**	Isorhamnetin	5000	Inactive	Active	Inactive
**5280343**	Quercetin	159	Inactive	Active	Active
**439246**	Naringenin	2000	Inactive	Active	Inactive
**5280863**	Kaempferol	3919	Inactive	Active	Inactive
**54454**	Simvastatin	1000	Inactive	Active	Inactive

Toxicological endpoints were predicted using ProTox 3.0. LD_{50}, median lethal dose; “Active” and “Inactive” indicate predicted presence or absence of the corresponding toxicological endpoint.
